# A rare mutation (p.F149del) of the *NT5C3A* gene is associated with pyrimidine 5′-nucleotidase deficiency

**DOI:** 10.1186/s11658-022-00405-w

**Published:** 2022-11-24

**Authors:** Dżamila M. Bogusławska, Michał Skulski, Rafał Bartoszewski, Beata Machnicka, Elżbieta Heger, Kazimierz Kuliczkowski, Aleksander F. Sikorski

**Affiliations:** 1 Department of Biotechnology, Institute of Biological Sciences, University of Zielona Góra, Prof. Z. Szafrana 1 St., 65-516 Zielona Góra, Poland; 2 Department of Cytobiochemistry, Faculty of Biotechnology, University of Wrocław, F. Joliot-Curie 14a St., 50-383 Wrocław, Poland; 3 Department of Biophysics, Faculty of Biotechnology, University of Wrocław, F. Joliot-Curie 14a St., 50-383 Wrocław, Poland; 4 Silesian Park of Medical Technology Kardio-Med Silesia, M. Curie-Skłodowskiej 10C St., 41-800 Zabrze, Poland; 5 Research and Development Centre, Regional Specialist Hospital, Kamieńskiego 73a St., 51-154 Wrocław, Poland

**Keywords:** Pyrimidine 5′-nucleotidase deficiency, Hereditary hemolytic anemia, Erythrocyte enzymopathy, Pyrimidine metabolism, Whole-exome sequencing

## Abstract

Pyrimidine 5′-nucleotidase deficiency is a rare erythrocyte enzymopathy. Here we report two cases of hemolytic anemia in brothers of Polish origin that are associated with a very rare mutation. Heterozygous deletion in the *NT5C3A* gene (c.444_446delGTT), inherited most likely from their asymptomatic mother, resulted in a single amino acid residue deletion (p.F149del) in cytosolic pyrimidine 5′-nucleotidase. However, only the mutated transcript was present in the reticulocyte transcriptome of both patients. Only residual activity of pyrimidine 5′-nucleotidase in the brothers’ erythrocytes could be observed when compared with the controls, including their asymptomatic father and sister. Western blot showed no sign of the presence of 5′-nucleotidase protein in the erythrocytes of both studied patients. The 2.5-fold reduction of the purine/pyrimidine ratio observed only in the brothers’ erythrocytes confirms the correlation of the results of molecular analysis, including whole-exome sequencing, with the phenotype of the pyrimidine 5′-nucleotidase deficiency. Altogether, our results may substantiate the hypothesis of the heterogeneity of the molecular basis of the defect involving both the mutation presented here and negative regulation of expression of the “normal” allele.

**Supplementary Information:**

The online version contains supplementary material available at 10.1186/s11658-022-00405-w.

## Introduction

Erythropoiesis in humans leads to the exclusive production of enucleated cells prior to entering the circulation, likely to improve the ability to traverse through capillaries [1, 2]. At the end of their approximately 100–120-day lifespan, macrophages of in the reticuloendothelial system, mainly in the spleen recognize the old erythrocytes to phagocytose them. Removal of erythrocytes in a normal mammalian organism is in balance with erythropoiesis, which is strictly controlled by several levels of physiological regulatory mechanisms, including extrinsic and intrinsic apoptotic pathways [3].

Hereditary hemolytic anemias (HHAs) are genetic disorders that present with anemia due to the increased destruction of circulating abnormal erythrocytes. Among them, hereditary spherocytosis (HS) is the most common in the Caucasian population [4, 5]. This group of diseases includes erythrocyte membranopathies, hemoglobinopathies, and enzymopathies [5–7]. The molecular defects are highly heterogeneous involving many different genes, and the severity of the disease varies widely, from fully compensated to transfusion-dependent anemia [4, 5, 8]. The molecular basis of numerous HHA subtypes has been solved; however, there are still a number of rare cases whose underlying molecular mechanisms are unexplored.

In contrast to membrane disorders, erythrocyte enzymopathies affect cellular metabolism but usually do not show the altered structural organization of the membrane [9–12]. Removal of the nucleus but also the mitochondria and RNA-containing ribosomes during enucleation and reticulocyte maturation [1] makes erythrocyte metabolism highly dependent on the anaerobic glycolysis, pentose phosphate pathway that produces reducing equivalents (NADPH) responsible for maintaining the reduced glutathione pool. Erythrocyte enzymopathies are inherited in an autosomal recessive manner, while compound heterozygotes are relatively rare, and only two are X-linked. They result in mild to severe hemolysis. The most common erythrocyte enzyme disorder is X-linked glucose-6-phosphate dehydrogenase deficiency (OMIM #300908). The remaining erythrocyte enzymopathies are much rarer and very often underdiagnosed. The least numerous and the rarest is the group of erythrocyte disorders involved in nucleotide metabolism. The defects are in three enzymes: pyrimidine 5′-nucleotidase encoded by the *NT5C3A* gene (cN-IIIA; P5N; OMIM #606224; 5′-nucleotidase, cytosolic IIIA; more than 60 families have been reported) as well as adenylate kinase and adenosine deaminase (purine metabolism; 12 and 3 affected families, respectively, have been reported) [9].

The only disorder affecting erythrocyte pyrimidine metabolism is pyrimidine 5′-nucleotidase deficiency (P5ND; OMIM #266120). A great majority of pyrimidines are removed during reticulocyte enucleation and maturation [9]. The removal of RNA degradation products from maturing reticulocytes is still required to prevent nucleotide accumulation. Pyrimidine 5′-nucleotidase is a relatively stable enzyme responsible for this process, which preferentially catalyzes the dephosphorylation of UMP and CMP [13–15]. The results of Chiarelli et al. show similar catalytic efficiency of cN-IIIA for both nucleosides [16]. This enzyme works synergistically with (deoxy)pyrimidine 5′(3′)-nucleotidase (cN-IIIB) encoded by the *NT5C* gene [12]. The action of these two enzymes allows nucleosides to passively diffuse out of the cell through the erythrocyte membrane, which is impossible for mononucleotides [14]. The activity of pyrimidine 5′-nucleotidase is much lower in mature erythrocytes than in reticulocytes. Interestingly, in contrast to other enzymes that depend on erythrocyte age, including hexokinase, aldolase, glucose-6-phosphate dehydrogenase, glutamic-oxaloacetic transaminase, and pyruvate kinase, a continuous decline in nucleotidase activity is observed during erythrocyte aging, not just in the first few days of maturation [17]. Substrate specificity of pyrimidine 5′-nucleotidase is not limited to UMP and CMP. Several other nucleoside 5′-monophosphates may also be accepted as substrates: m7GMP, AZT monophosphate, AraC monophosphate, dCMP, dTMP, and dUMP [18, 19].

Although, as suggested by Hirono et al., cN-IIIA deficiency is the third most common erythrocyte enzymopathy, the disease is diagnosed very rarely [20]. Clinical symptoms of the disease are usually characterized by anemia, jaundice, hemoglobinuria, hepato- and splenomegaly, hyperbilirubinemia, and reticulocytosis. In contrast to erythrocyte membranopathies, osmotic fragility is ordinarily not increased [21]. Despite the fact that no known natural mutation is directly involved in the catalytic mechanism, a significant reduction in enzyme activity is observed in patients with P5ND [12, 13, 15, 22, 23]. The exact mechanisms leading to erythrocyte destruction and hemolytic anemia are still unclear, but it is known that pyrimidine nucleotide accumulation affects erythrocyte metabolism, including the decrease in intracellular pH, lower G6PD activity, and reduced ribose-phosphate pyrophosphokinase activity [12, 15, 24–26].

**Fig. 1:**
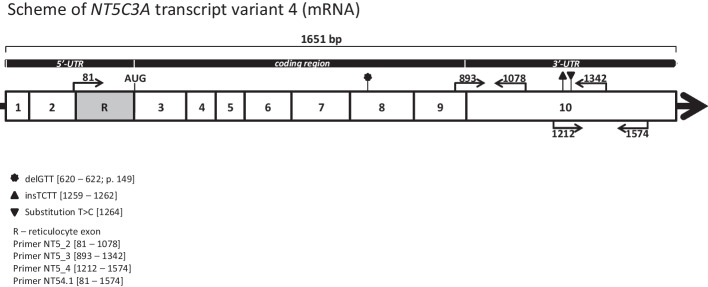
Scheme of the *NT5C3A* gene transcript variant 4 (NM_001002010.2)—erythrocyte-specific nucleotidase isoforms (cN-IIIA-R). Additional exon R appears only in the reticulocyte mRNA splice variant, which was detected downstream of exon 2 [28]. The figure shows the location of the in-frame deletion (p.F149del) detected in patients with HA in the studied family. Localization of the primers used for cDNA amplification is indicated by arrows

As mentioned above, although transcriptomic and functional studies have shown that the *NT5C3A* gene is expressed in various tissues and cell lines, only one hereditary disease (P5ND) is correlated with mutations in this gene [27, 28], which is most likely a consequence of very limited erythrocyte metabolism and expression of erythrocyte-specific nucleotidase isoform (cN-IIIA-R). As Kanno et al. proved, an additional exon R appears only in the reticulocyte mRNA splice variant, downstream of exon 2 [28]. The start codon is located at the beginning of exon 3 (Fig. 1). Interestingly, the exon R is not detected in erythroleukemic cell lines or bone marrow. Moreover, as shown by qPCR, the cN-IIIA-R isoform (285 amino acid residues) is dominantly expressed in reticulocytes. Although two more nucleotidase isoforms, p36 and P5N-I (286 and 297 amino acid residues, respectively), are also detected in reticulocytes, they do not contain the key exon and their biological function seems to be marginal [28].

During our studies on HS cases in the Polish population [29–31], we identified erythrocyte membrane protein abnormalities in the studied family in which two members (brothers, RK and MK) displayed symptoms of hemolytic anemia that did not fit the characteristics of the known disease [32]. We used high-throughput sequencing technologies and functional studies to characterize the molecular basis of this anemia case. All presented data confirm the correlation of the results of the molecular analysis with the phenotype of pyrimidine 5′-nucleotidase deficiency.

## Materials and methods

### Patients

During our studies, we identified a family in which two members (brothers, RK, MK) displayed symptoms of hemolytic anemia that did not match the characteristics of the known disease [32]. Two brothers with symptoms of hemolytic anemia were admitted to the out-patient Clinic of the Department of Haematology, Blood Neoplasms and Bone Marrow Transplantation, Wroclaw Medical University. This study was approved by the ethics committee (permission KB-541/2011) of the Wroclaw Medical University, and informed consent was obtained from the patients and healthy patients’ family members and unrelated healthy individuals serving as a controls.

The brothers were hospitalized as children at the age of 6 and 4 years (RK and MK, respectively) with an initial diagnosis of hereditary spherocytosis (HS). Further studies ruled out HS, but the conclusive and accurate diagnosis was not possible. Diagnostic criteria for HA were based on features: yellow skin and a high hard palate, splenomegaly, jaundice, increased bilirubin, reticulocytosis, and anemia (Table 1). Both patients had normal values of erythrocyte osmotic fragility and presence of stomatocytes on peripheral blood smears (as well as anisocytosis and azurophilic stipplings) but no spherocytes typical of hereditary spherocytosis in their blood smears. The EMA-binding test, a reliable method for identifying hereditary spherocytosis showed an increase in mean fluorescent intensity (109–112% of reference), whereas for typical spherocytosis a decrease is observed [33, 34]. The direct anti-globulin test was negative, and other causes of hemolytic anemia such as thalassemia syndromes and G6PD deficiency were excluded by appropriate tests (Additional file 1: Table S1.1). SDS–PAGE profile of their erythrocyte membrane protein was not different from normal [35], as was Na^+^K^+^ equilibrium of the erythrocytes.

**Table 1:** Hematological characteristics of studied family members

Studied members of the family		Age (years)	RBC (T/L)	HCT (L/L)	Hb (mmol/L)	Total bilirubin (µmol/L)	Ret (fraction)	EMA test (MFI)*
References for male		Adults	4.2–6.0	0.40–0.54	8.69–11.17	3.42–22.24	0.005–0.015	–
References for female		Adults	4.0–5.0	0.37–0.47	7.54–9.93	3.42–22.24	0.005–0.015	–
Asymptomatic family members	EK (female)	31	4.92	0.43	9.18	8.21	0.020	ND
	AK (male)	61	4.70	0.44	9.62	8.04	0.007	ND
Patients with HA	RK (male)	41	3.43 ± 0.12	0.34 ± 0.01	7.08 ± 0.21	32.07 ± 3.02	0.048 ± 0.029	5.73 (ref. 5.1 ± 0.4)
	MK (male)	36	3.36 ± 0.15	0.30 ± 0.01	6.14 ± 0.16	111.23 ± 27.70	0.065 ± 0.021	114.05 (ref. 98.31–110.23)

RBC, erythrocytes; HCT, hematocrit; Hb, hemoglobin; Ret, reticulocytes; MFI, mean fluorescence intensity; ND, not determined. *The analyses were performed by two independent laboratories

### DNA and RNA isolation

EDTA anticoagulated blood was collected from two patients (brothers, RK and MK) who displayed symptoms of hemolytic anemia and asymptomatic family members (father AK and sister EK) as well as volunteers (healthy individuals of Polish origin), and a patient with HS [29, 32] by venipuncture. The genetic material of a likely asymptomatic mother of the brothers was unavailable. Genomic DNA was isolated from the whole blood of each studied family member using the standard method (QIAamp DNA Blood Mini Kit, Qiagen, Hilden, Germany) and stored at −20 °C until analysis. Total RNA was extracted from whole blood as well as reticulocytes and collected using the miRNeasy Mini Kit (Qiagen, Hilden, Germany). Both procedures were carried out according to the manufacturer’s recommendations. RNA samples were stored at −70 °C until use. DNA and RNA concentrations were calculated on the basis of the absorbance at 260 nm.

### Transcriptome analysis (RNA-seq)

Transcriptome analysis was performed by the Heflin Center for Genomic Science Core Laboratories, the University of Alabama at Birmingham, AL, USA. Reticulocyte RNA samples isolated from patients with HA and two controls [one healthy control (CtrlH) and one affected HS [29, 32]] were sequenced and aligned to the human reference genome assembly (human genome19). For contextualization of expression patterns and functional signatures, the RNA-seq results were analyzed using the GeneAnalytics tool [36]. The full procedure has been described by Skulski et al. [32].

### Whole-exome sequencing on Illumina platforms

Exome sequencing was performed for both affected patients as well as their father and sister. DNA samples were sequenced at the Heflin Center, UAB (see above). Exome capture was carried out using the Agilent SureSelect all Human exon v3 capture kit (Agilent SureSelect Human All Exon 50 Mb for target enrichment; Agilent Technologies, Santa Clara, CA, USA). The SureSelect Target Enrichment System (Agilent Technologies, CA, USA) was used, followed by 2 × 100 bp paired-end sequencing on the HiSeq 2000 next generation sequencer from Illumina (Illumina, San Diego, CA, USA). Raw sequence reads were aligned to the reference human genome (human genome19/GRCh37.13). The bioinformatics analysis for detecting single nucleotide variants (SNVs) and inserts/deletions was performed at the Heflin Center at UAB and annotated according to dbSNP*.*

### Whole-exome sequencing data analysis

Trimmed raw data were aligned to the hg19 human reference genome, and variant identification analysis was performed using the Ingenuity Variant Analysis plugin (IVA; QIAGEN, CA, USA). Additionally, variants that may be responsible for the observed phenotype were also made to select compound polymorphisms present in one of the genes or several genes (connected by common metabolic pathways) using the GeneAnalytics tool (LifeMap Sciences, CA, USA; accessed on 22 December 2016). The clinical significance of selected changes was made according to the 1000 Genome Browser [37], ClinVar [38], HGMD [39], Online Mendelian Inheritance in Man (OMIM) [40], Single Nucleotide Polymorphism (dbSNP) [41], GeneCards [42], and The Universal Protein Resource (UniProt) [43] databases (accessed on 29 December 2020) as well as literature data.

### Sanger sequencing validation

Potentially pathogenically significant variants identified by WES were verified by target Sanger sequencing of genomic DNA and cDNA. Total RNA was reversely transcribed into cDNA for sequencing using the RevertAid First Strand cDNA Synthesis Kit (Thermo Fisher Scientific, Waltham, MA, USA) according to the manufacturer’s recommendations. Sanger sequencing and all primer synthesis were performed by Genomed SA (Warsaw, Poland). The sequences of all primers and the PCR amplification parameters are available upon request. Additional file 1: Tables S1.2 and S1.3 include the sequences of the primers used for verification of the key data obtained from WES.

### SDS–PAGE and western blotting

After freezing and thawing five times in Red Cell Stock Solution [0.9% (w/v) NaCl containing 28% (w/v) glycerol and 2.8% (w/v) sorbitol], erythrocyte lysate was centrifuged at 10,000*g* for 5 min at 4 °C, and the supernatant was collected. Equal volumes of supernatant and reducing agent [40 mM Tris–HCl, 1.5% (w/v) SDS, 1% (w/v) 2-mercaptoethanol, 1.3 M urea, and 0.005% (w/v) bromophenol blue] were mixed and incubated for 5 min at 95 °C for solubilization and then centrifuged at 10,000*g*, for 5 min at 4 °C. Supernatants were mixed with reducing agent in a ratio of 1:4 and loaded on (12% separating, 4.5% stacking) polyacrylamide gel. Electrophoresis was carried out at a constant current of 25 mA for 2 h at room temperature in a running buffer [0.025 M Tris, 0.192 M glycine buffer, pH 8.3 containing, and 0.1% (w/v) SDS] until bromophenol blue reached the bottom of the gel. After electrophoresis, a wet transfer onto nitrocellulose paper was performed at a constant current of 250 mA in transfer [Towbin buffer [44]—25 mM Tris buffer, pH 8.3 containing 0.192 M glycine and 20% (v/v) methanol] for 2 h.

Then membranes were stained with Ponceau S solution [0.2% (w/v) Ponceau S and 5% (v/v) acetic acid] to confirm proper protein transfer. Membranes were blocked in 5% nonfat dry milk solution in TBS-T buffer [20 mM Tris–HCl buffer, pH 7.5 containing 0.15 M NaCl and 0.05% (v/v) Tween-20] for 45 min with gentle shaking at room temperature. After washing with TBS buffer (20 mM Tris buffer, pH 7.5 containing 0.15 M NaCl), membranes were incubated with the primary, mouse, monoclonal antibody anti-NT5C3 (sc-390782, Santa Cruz Biotechnology, Dallas, TX, USA), diluted 1:2000 in TBS-T buffer, for 2 h at room temperature with gentle shaking. Then, after washing the membrane in TBS-T buffer, a secondary goat-anti-mouse IgG (H + L) antibody, conjugated with HRP (Jackson ImmunoResearch, Ely, UK), was used to detect the primary antibody. Nitrocellulose membranes were incubated with secondary antibody diluted 1:10,000 in TBS-T buffer for 60 min in the dark at room temperature with gentle shaking. After washing the membrane in TBS-T buffer a chemiluminescence reaction was induced by using ECL Western Blotting Detection Reagent (RPN 2209, Amersham, Marlborough, MA, USA) and visualized in the Azure c500 Imaging System (Azure Biosystems, Dublin, CA, USA). Quantification of WB membrane by ImageJ 1.51n software was performed according to the procedure described by Stael et al. [45]. The area of each peak (without background) for relative density calculation was used.

### Enzymatic activity

The estimation of enzymatic activity of cytosolic pyrimidine 5′-nucleotidase was performed according to Beutler et al. (1984) with slight modifications [46]. The whole blood was collected by venipuncture using EDTA as an anticoagulant, diluted 1:1.5 with cold 0.9% NaCl and passed through a leukofilter as described previously [32]. The flow-through was centrifuged (600*g*, 15 min, 4 °C) and then the erythrocyte pellet was washed three times (800*g*, 10 min, 4 °C) with cold 0.9% NaCl. Erythrocyte pellets were suspended in Red Cell Stock Solution (mentioned in the above paragraph) in a ratio of 1:1 (v/v) and lysed via five freezing and thawing cycles. The UMP (U6375, Sigma-Aldrich, Saint Louis, MO, USA) as a substrate for P-5′-nucleotidase was dissolved in a buffer (0.1 M Tris–HCl, 0.1 M MgCl_2_, 0.1 M EDTA, and 0.7 mM 2-mercaptoethanol) to a final concentration of 4.6 mM. Next, 150 µL of hemolysate (diluted 1.25- to 5-fold) and 150 µL of UMP solution were mixed and incubated at 37 °C for 120 min. Then 120 µL of the mixture was transferred to 1200 µL of iron–TCA solution (10% trichloroacetic acid, 1% thiourea, 3% ammonium iron (II) sulfate hexahydrate) to stop the reaction. The control sample was prepared by taking 120 µL of incubation mixture immediately after mixing 150 µL of hemolysate and 150 µL of UMP solution and diluting in 1200 µL of iron–TCA solution. Samples were centrifuged (10,000*g*, 5 min, RT), and the supernatants (FeTCA extracts) were collected in clean tubes. Inorganic phosphate (P_i_) was quantitated by the Rouser method in a 96-well plate: 120 µL of each FeTCA extract, 20 µL of 70% perchloric acid, 30 µL of dH_2_O, 20 µL of 2.5% (w/v) ammonium molybdate, and 20 µL of 10% (w/v) ascorbic acid were added [47]. As a blank sample, dH_2_O was used in place of acid extract. The plate was gently shaken on the orbital shaker and later incubated for 30 min at 37 °C. After incubation, absorbance at 750 nm was read on the Rayto RT-6100 microplate reader (Rayto Life and Analytical Sciences, Shenzhen, China). On the basis of the calibration curve, the P_i_ nanomolar concentration in each sample was calculated. To convert these data into specific enzymatic activity (expressed as pmol P_i_/mg Hb/min), hemoglobin was quantified using absorbance at 512 nm and the molar extinction coefficient for hemoglobin of 26,936.8 cm^−1^/M [48] (accessed on 4 July 2022). Nonspecific nucleotidase activity tested on 5′-AMP as a substrate showed low values in the range of 0–15% (data not shown). Each sample was accompanied by the zero time control, thus including “background” phosphate derived from the sample and from the 5′-UMP.

### Measurement of miRNA and mRNA levels using qPCR

The StepOnePlus Real-Time PCR System was used to run the qPCR reactions. TaqMan One-Step RT-PCR Master Mix Reagents (Thermo Fisher Scientific, Waltham, MA, USA) and the manufacturer’s protocol were used as described previously [49]. The relative expression levels were calculated using the comparative relative standard curve method [50]. We used glyceraldehyde-3-phosphate dehydrogenase (GAPDH) mRNA and small nucleolar RNA (C/D Box 44 (*RNU44*) RNA) as the relative control for our studies. The relative control stability during experiments was verified with 18S rRNA. TaqMan probes used were the following: *GAPDH*, Hs99999905_m1; *RNU44*, 001094; 18 s, Hs99999901_s; 5′-nucleotidase, cytosolic IIIA (*NT5C3A*, transcript variant 4 expressed only in reticulocytes), Hs01052076_m1 and Hs01052075_m1; zinc finger DHHC-type containing 17 (*ZDHHC17*), *Homo sapiens* microRNA 4775, 463622_mat.

### Molecular karyotype (array CGH)

Identification of genome dosage imbalances with the array CGH method (the array-based Comparative Genomic Hybridization) was performed in a certified laboratory cooperating with the Genomed Healthcare Center, Warsaw, Poland (The Institute of Mother and Child, Warsaw, Poland). Material collected from two family members was analyzed: patient RK (analysis of gDNA isolated from saliva, the Oragene kit, DNA Genoteck) and the asymptomatic father AK (analysis of gDNA isolated from peripheral blood collected in an EDTA Vacutainer).

### Luciferase reporter assays

Assignment of potentially important microRNAs predicted to change the mRNA expression levels of the *NT5C3A* gene was performed using miRBase [51]. Human 3′ untranslated region (UTR) of the wild type of the *NT5C3A* gene (WT -HmiT067108-MT06) and three different mutation variants (M1 with substitution T/C, M2 insertion TCTT, and M1 with both substitution T/C and insertion TCTT; for details, see Additional file 1: Fig. S1.1) of firefly luciferase reporter constructs (HmiT018551-MT06-01/02/03, respectively) and their control vector (Vc) (CmiT000001-MT06) were purchased from GeneCopoeia (Rockville, MD, USA). The post-transcriptional activity of the 3′-UTR regions of the human erythrocyte-specific nucleotidase isoform (P5N-R) was tested by transfecting HEK293T cells with the constructs above described or with control plasmid. Cells were seeded into a 24-well plate and incubated for 24 h at 37 °C until 80% confluency. Cells were transfected with expression plasmids using Lipofectamine 2000 (Thermo Fisher Scientific) according to the manufacturer’s instructions. Each well received 300 ng of total plasmid DNA (control vector or WT or mutated sequences M1, M2, M3) as well as vectors: *hsa-miR-4775 mimic* (MC21381, Thermo Fisher Scientific) or *hsa-miR-4775 inhibitor* (AM21381, Thermo Fisher Scientific) or hsa-miR-4775 precursor (PM21381, Thermo Fisher Scientific) or the cel-miR-67 scramble control (Thermo Fisher Scientific) at a final concentration of 15 pM. After 48 h of transfection cells were lysed and dual-luciferase reporter assay was performed according to the manufacturer’s protocol (Promega, Madison, WI, USA). Firefly luciferase expression values were normalized to Renilla luciferase expression values. Standard deviations were calculated from independent biological and technical replicates. Results were plotted as a relative decrease in arbitrary light units compared with control cells.

### Determination of the pattern of methylation

Peripheral blood samples of four studied family members and five Polish healthy subjects were obtained under the approval of the Ethics Committee of Wroclaw Medical University (study protocol KB-199/2017). DNA was extracted from a 200 µL whole blood sample using QIAamp DNA Blood Mini Kit (Qiagen, Hilden, Germany) following the instructions of the manufacturer. The bisulfite sequencing PCR method (BSP) was used for methylation mapping. EpiJET Bisulfite Conversion Kit (Thermo Fisher Scientific) was used in nine samples using 180–400 ng of gDNA. The CpG-rich amplified fragments of the *NT5C3A* gene, after the bisulfite conversion of unmethylated cytosines, are then sequenced. PCR amplicons were used for bisulfite conversion and Sanger sequencing. Primer homologous to the converted (NT5C3A_bsDN) and unconverted template (NT5C3A_gDNA) were used for detecting converted amplicons. Primer homologous to the unconverted template (gDNA) were used for amplification of gDNA (Additional file 1: Table S1.4). Primers detecting converted template (NT5C3A_bsDNA) did not include cytosines located within the CpG islands (Additional file 1: Fig. S1.2A). They had homology only to the converted cytosines.

### Statistical analysis

GraphPad Prism v. 6.01 and MS Excel software was used to process all data in this work, presented as mean ± standard deviation. Comparisons were performed using Student’s *t*-test. Statistical significance was accepted as a *p*-value of < 0.05, used for most analyses besides next-generation sequencing (NGS) analysis where the false discovery rate (FDR)-corrected *p*-value, i.e., *q*-value, of < 0.05 was considered as significant [52].

## Results

Basic as well as additional clinical and hematological data for patients with HA (brothers RK and MK) and two asymptomatic family members, the father (AK) and sister (EK), are summarized in Table 1 and Additional file 1: Table S1.1 and S1.5. First a preliminary transcriptome analysis was carried out for both brothers with HA and two controls, one healthy and one with HS. The latter was chosen owing to high reticulocytosis [29] and presence of immature reticulocytes (Heilmeyer group II and III) in the peripheral blood.

### Analysis of RNA-sequencing data

The RNA-seq data were then analyzed by Cufflinks, and results for each patient with HA were compared independently with both controls. Statistically significant differences (*p* < 0.05) in gene expression were detected for only nine transcripts: *ATF3*, *CDK1*, *CHEK1*, *CLDN6*, *APOE*, *NOP56*, *UBE2E1*, *MEST*, and *NGFRAP1*, whose expression was decreased only for patient RK versus the healthy control (CtrlH) (Additional file 2, Additional file 4). None of these genes encodes proteins functionally related to the HA phenotype [32].

### Validation of whole-exome sequencing data

Whole-exome sequencing (WES) was carried out twice for each sample and included all available members of the studied family: both brothers with symptoms of HA (RK and MK) and two asymptomatic family members, i.e., the father (AK) and sister (EK). Maternal genetic material was unavailable. Quality control of WES raw reads is presented in Additional file 1: Table S1.6. Data verification was also performed, using two bioinformatics tools: Ingenuity Variant Analysis and GeneAnalytics tools. The searches were carried out in several stages (Additional file 1: Fig. S1.3).

In total, 27,460 nucleotide sequence changes present in the genetic material of both brothers were detected, of which 729 known mutations/polymorphisms found in the Human Genome Mutation Database have been identified. However, only 365 are present in either of the brothers and do not appear to be related to the observed symptoms of hemolytic anemia. We focused on variants with potentially large effects (missense, nonsense, splicing site, and frameshift), which allowed us to select 2533 variants (Additional file 1: Table S1.7). Particularly interesting for us were genes associated with clinical phenotypes of known inherited hemolytic anemia [6], including erythrocyte membranopathies (*ANK1*, *SPTB*, *SPTA1*, *SLC4A1*, *EPB42*, *EPB41*, *PIEZO1*, *KCNN4*, *RHAG*), erythrocyte enzymopathies (*G6PD*, *PKLR*, *ENO1*, *AK1*, *GPI*, *NT5C3A*, *GCLC*, *GPX1*, *GSR*, *GSS*, *HK1*, *BPGM*, *PGK1*, *TPI1*), and erythrocyte hemoglobinopathies (*HBB*, *HBA1*, *HBA2*). Known polymorphisms have been detected in 14 genes involved in known erythrocyte pathologies, including 8 functional variants with potentially large pathological effects (Additional file 1: Table S1.8).

Further, homozygous polymorphisms present in both brothers were also checked, which at the same time occurred in a heterozygous state and/or were absent in other family members or were located in the X chromosome. In this way, we selected over 544 polymorphisms, which were analyzed mainly according to the functions of the encoded proteins. An important factor was low frequency in the 1000 Genomes and/or ExAC datasets (up to 0.5%), which reduced the list to 15 variants. Another approach was to verify the variants present in both brothers on the basis of low frequency regardless of the inheritance pattern. In the pool of variants whose current frequency did not exceed 0.5% (according to the above-mentioned datasets), 830 changes were detected in both brothers.

A complementary approach was the selection of variants localized in genes expressed in erythroid cells such as CD71^++++^ and/or GPA^++^ type cells. Nucleotide sequence changes obtained from WES data were compared with the data deposited in the UniGene NCBI database: CD71^++++^ library (Lib,8975) and GPA^++^ library (Lib,11923). These data allowed us to select 701 and 654 genes deposited respectively in the above-mentioned libraries representing the early and late reticulocyte maturation stages, while 201 genes are common to both libraries (Additional file 1: Table S1.9).

Validation of changes in gDNA using Sanger sequencing was carried out for 65 polymorphisms located in 46 genes. They were selected on the basis of the functions performed by the proteins encoded by these genes, their frequency, and inheritance pattern. Sequence changes that were not confirmed by Sanger method were removed from the list. The polymorphism rs750339397 has been corrected (Additional file 1: Table S1.10).

We also attempted to identify the HA variants important for the phenotypes using the Ingenuity Variant Analysis bioinformatics tool (Qiagen Hilden, Germany). The summary of data obtained this way is presented in Additional file 1: Table S1.11. Among three particular changes in the course of selection using this tool including the biological context, the following were listed: heterozygous missense mutation of the *DNAH11* gene (V2924M, rs72657369), heterozygous frameshift mutation of the *NT5C3A* gene (p.F149del, rs1227859962), and homozygous missense mutation of the *HCFC1* gene (p.L1587F, rs1557112939). These variants were detected only in the studied patients with HA.

Finally, the verification of inheritance patterns and the frequency of occurrence of polymorphisms constantly updated during the project execution limited the pool of genes that today seem to be important for the phenotype. All strategies used have identified 12 rare mutations in different genes that might be responsible for the observed phenotype in the studied family (Table 2). Information on each gene coming from the GeneCards database in which these variants were detected, including the gene name and known functions/pathways of normal and pathological gene products, is presented in Table 3.

**Table 2:** Genetic variants that might be responsible for the observed phenotype in the studied family

Gene name/chromosome	SNP Reference number	Frequency of change* MAF/minor Allele count	Change of nucleotide/amino acid residue HGVS names	Inheritance patients/asymptomatic family members (WES)				Clinical significance ClinVar NCBI
				RK	MK	EK	AK	
***NT5C3A*** chr7	rs1227859962	delACA = 0.000014 (2/140214, GnomAD) delACA = 0.00007 (1/15150, ALFA) delACA = 0.0002 (1/4480, Estonian)	NM_001002010.2:c.597_599delGTT NP_001361265.1:**p.Phe149del**	het	het	abs	abs	Not reported
***TYMP*** chr22 and ***SCO2*** chr22	rs11479	A = 0.087629 (12223/139486, ALFA) A = 0.1428 (715/5008, 1000G) A = 0.1105 (495/4480, Estonian)	***TYMP*** NM_001113755.2:c.1412C > T NP_001107227.1:**p.Ser471Leu** ***SCO2*** (nearGene-5) NM_005138.2:c.-349C > T	het	het	het	abs	Benign (10 July 2021)
	rs112723255	T = 0.045881 (10439/227524, GnomAD_exome) T = 0.04446 (2148/48310, ALFA) T = 0.0569 (255/4480, Estonian)	***TYMP*** NM_001113755.2:c.1393G > A NP_001107227.1:**p.Ala465Thr** ***SCO2*** (nearGene-5) NM_005138.2:c.-368G > A	het	het	abs	het	Benign/ Likely benign (31 July 2021)
***PUDP*** chrX	rs187333600	A = 0.014659 (3880/264690, TOPMED) A = 0.018758 (1934/103104, GnomAD) A = 0.02251 (615/27324, ALFA)	UTR-5 NM_001135565.1:c.-14C > T	hom	hom	het	abs	Not reported
***HCFC1*** chrX	rs1557112939	None	NM_005334.2:c.4759T > C NP_005325.2:**p.Leu1587Phe**	hom	hom	abs	abs	Uncertain significance (24 April 2017)
***GEMIN8*** chrX	rs145874697	T = 0.000638 (169/264690, TOPMED) T = 0.000769 (141/183241, GnomAD_exome) T = 0.000987 (104/105340, ALFA)	NM_001042480.1:c.514G > A NP_001035945.1:**p.Val172Met**	hom	hom	het	abs	Not reported
***POLE*** chr12	rs5745066	T = 0.018814 (5004/265972, ALFA) T = 0.014164 (1986/140212, GnomAD) T = 0.0201 (90/4480, Estonian)	NM_006231.3:c.6418G > A NP_006222.2:**p.Glu2140Lys**	hom	hom	abs	het	Benign/ Likely benign (8 December 2020)
***XK*** chrX	rs2230148	T = 0.169863 (44961/264690, TOPMED) T = 0.167078 (17326/103700, GnomAD) T = 0.17009 (3524/20718, ALFA)	NM_021083.3:c.*89A > T	hom	hom	het	abs	Not reported
***RAD51C*** chr17	rs28363317	G = 0.005602 (1407/251152, GnomAD_exome) G = 0.006531 (916/140262, GnomAD) G = 0.0063 (28/4480, Estonian)	NM_058216.1:c.859A > G NP_478123.1:**p.Thr287Ala**	het	het	abs	abs	Benign/ Likely benign (1 February 2021)

Gene names and missense mutations are shown in bold

According to NCBI; het, heterozygotic; hom, homozygotic; abs, absence. *Accessed on 16 November 2021

**Table 3:** Known functions/pathways of a normal and pathological gene product of affected genes

Gene name	Known functions/pathways of normal and pathological gene products of affected genes that are potentially important for the HA phenotype in the studied family
*NT5C3A*	Pyrimidine and purine metabolism (REACTOME, KEGG); catalyze the dephosphorylation of nucleoside 5′-monophosphates; mutations in this gene are a cause of hemolytic anemia due to uridine 5′-monophosphate hydrolase deficiency; An important paralog of this gene is *NT5C3B*; expression in erythroblasts and CD71^++++^ cells
*TYMP*	Pyrimidine metabolism (REACTOME, KEGG); catalyzes the reversible phosphorolysis of thymidine; predicted interaction of *TYMP* and *SCO2* genes products; expression in erythroblasts
*SCO2*	Pyrimidine deoxyribonucleoside degradation; predicted interaction of *TYMP* and SCO2 gene products; expression in erythroblasts
*PUDP*	Pyrimidine metabolism (REACTOME, KEGG); dephosphorylates pseudouridine 5-phosphate, a potential intermediate in rRNA degradation, and pseudouridine is then excreted intact in urine; expression in erythroblasts
*HCFC1*	Host cell factor; diseases associated with *HCFC1* include methylmalonic acidemia and homocysteinemia (inherited in an X-linked manner); expression in erythroblasts
*GEMIN8*	mRNA processing; expression in erythroblasts
*POLE*	2′-Deoxyribonucleoside-5′-triphosphate: DNA deoxynucleotidyltransferase; participates in DNA repair and chromosomal DNA replication; expression in erythroblasts
*XK*	The kx blood-group antigen (kxa) family; diseases associated with XK include Mcleod neuroacanthocytosis syndrome and choreoacanthocytosis; expression in erythroblast and GPA^++^
*RAD51C*	Fanconi anemia group O, expression in erythroblasts and GPA^++^ cells

Developed based on data collected in the GeneCards database. Expression of the genes in erythroblasts [53]; erythroid cell lines and/or CD71^++++^ and GPA^++^ type cells [based on the GeneCards Database and UniGene NCBI database: CD71^++++^ library (Lib,8975) and GPA^++^ library (Lib,11923)]

Analyses of the data collected at this stage, the biological functions of the proteins encoded by these genes, and the results on the expression level of these genes currently do not allow us to assume that only one mutation is responsible for the phenotypic effect. Using the GeneAnalytics web server, the results of WES analyses were convincingly assigned to 20 metabolic pathways (with the highest score) (Additional file 1: Table S1.12). Importantly, our set of gene transcripts was associated with four phenotypes that correlate with purine and pyrimidine metabolism. Polymorphisms/mutations in four genes coding for the proteins responsible for purine and pyrimidine metabolism were identified in this way (*NT5C3A*, *TYMP*, *SCO2*, and *PUDP*).

**Fig. 2:**
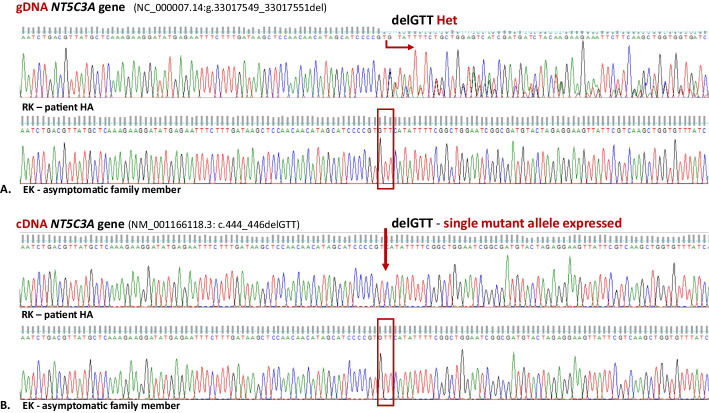
The rare *NT5C3A* gene mutation (rs1227859962) is associated with pyrimidine 5′-nucleotidase deficiency. Fragment of sequencing traces of the *NT5C3A* gene in an affected patient (RK) and asymptomatic family member (EK). The gDNA sequence analysis (**A**) revealed a heterozygous deletion (NC_000007.14:g.33017549_33017551del) only in both affected patients in contrast to cDNA sequence analysis (**B**), which revealed only a single mutant allele with a deletion (NM_001166118.3: c.444_446delGTT) causing the single amino acid deletion (NP_001361265.1:p.Phe149del) in cytosolic pyrimidine 5′-nucleotidase

In the next step of the project, we verified the presence of potentially key variants in the cDNA. Validation of changes in cDNA using Sanger sequencing was carried out for 15 genes (Additional file 1: Table S1.13). Only in the case of the *NT5C3A* gene did the obtained results suggest potential significance for the phenotype. In this case, the analysis was carried out in two stages using nested PCR. The outer primers were used to amplify the 1493 bp fragment that was used to amplify all known variants of the transcripts for the *NT5C3A* gene. In the next step, the internal primers allowed the amplification of the only fragment of the reticulocyte mRNA splice variant cN-IIIA-R (Fig. 1). Its sequence was not complementary to that of the pseudogenes, the presence of which was pointed out by other authors [54]. This allowed reliable information to be obtained on the sequence of the dominant isoform in the reticulocytes of patients and their family members. Only the presence of a mutated allele (p.F149del, rs1227859962) for both brothers was detected. Again, this change was previously revealed to be heterozygous in WES (Fig. 2 and Additional file 1: Fig. S1.4). This change was not detected in the gDNA and cDNA of the father and sister.

### Functional analysis

Standards and guidelines for the interpretation of sequence variants including recommendations for the use of specific standard terminology are currently a huge challenge due to the huge amount of data deposited in various databases. The use of highly advanced bioinformatics tools in such analysis is an extremely demanding task. In-frame deletion (p.F149del, rs12278599) discovered through WES was automatically classified by using Ingenuity Variant Analysis plugin results (QIAGEN, CA, USA) based on the American College of Medical Genetics and Genomics and the Association for Molecular Pathology Guidelines [55] as a variant of likely pathogenic (Additional file 3): “Criteria for classifying p.F149 del as likely pathogenic variants: PM1: Located in a critical and well-established functional domain (UMPH-1) without benign variation (Moderate): PM2: Absent from controls (or at extremely low frequency if recessive) in GnomAD (the Genome Aggregation Database), in these sources of population frequency data, this variant frequency falls below 1% for this recessive phenotype (Moderate); PM4: Protein length change as a result of an in-frame deletion/insertion in a non-repeat region (Moderate). Evidence against pathogenicity: none.”

The following functional consequences due to the p.F149del mutation using the Ingenuity Variant Analysis plugin (QIAGEN, CA, USA) were predicted: CADD score 18.490 (likely deleterious) and conservation phyloP *p*-value 1.230 × 10^−5^ (highly conserved) (Additional file 3). Detecting only the mutated variant of the transcript in both affected patients should result in biological changes, which we have tried to describe in the next steps.

### Gene expression analysis

**Fig. 3:**
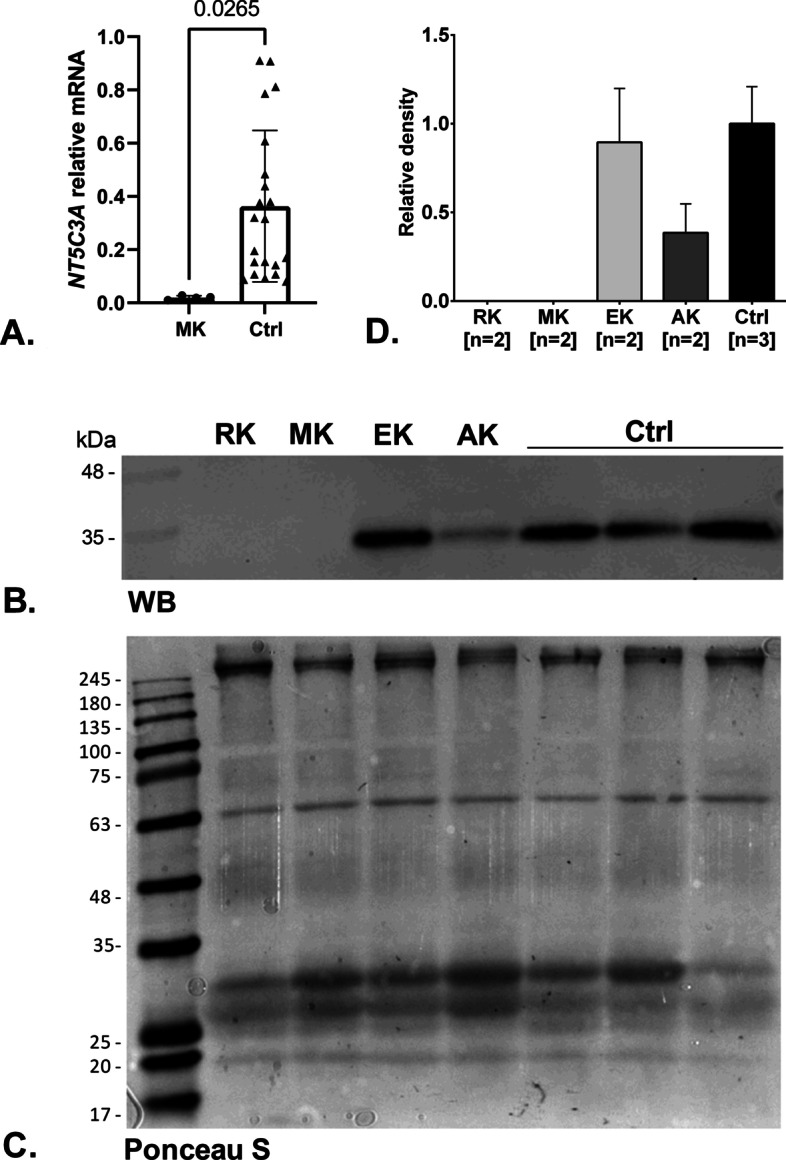
Functional analysis. **A** The *NT5C3A* gene expression analysis by quantitative PCR showed a very low expression level in patient MK compared with controls (healthy individuals). Error bars represent the standard deviation. **B** Western blot analysis of cytosolic pyrimidine 5′-nucleotidase expressed in erythrocyte (RK and MK—patients with HA, AK—asymptomatic father, EK—asymptomatic sister, and controls—healthy subjects). The volume corresponding to equal protein quantity of loaded samples is 25 µL. **C** Ponceau-S-stained nitrocellulose membrane as an alternative loading control. **D** Densitometric analysis of western blot assays for cytosolic pyrimidine 5′-nucleotidase present in the erythrocyte. The area of each peak (without background) for relative density calculation was used. Error bars represent range. Samples of all studied family members correspond to technical replicates (*n* = 2), while control samples correspond to biological replicates (*n* = 3)

We started gene expression analysis with the expression level of the reticulocyte mRNA splice variant cN-IIIA-R. It was performed using RNA isolated from reticulocytes of patient MK and control samples (healthy individuals). The *NT5C3A* gene expression analysis performed by quantitative PCR showed a very low level of 5′-nucleotidase expression compared with the controls (Fig. 3A).

Western blotting analysis was preformed to check the proper translation of cytosolic pyrimidine 5′-nucleotidase from mRNA. The monoclonal anti-NT5C3 antibody was used to detect nucleotidase protein. Immunoblotting showed no detectable signal for both RK and MK patients (Fig. 3B). Moreover, a band corresponding to expressed protein in the AK sample (asymptomatic father) was weaker (~ 60%) than in the control samples, probably owing to downregulation of the expression, which, however, did not cause a substantial decrease in enzyme activity (see below) and remained without clinical implications. Owing to the similar molecular weight between P-5′-nucleotidase and actin beta as an alternative loading control, the Ponceau-S-stained membrane was used (Fig. 3C). Quantification of WB membrane confirmed that in both patients (RK and MK) protein was not detected, while a significant decrease was observed in the father (Fig. 3D).

### Enzymatic activity and purine/pyrimidine ratio

**Fig. 4:**
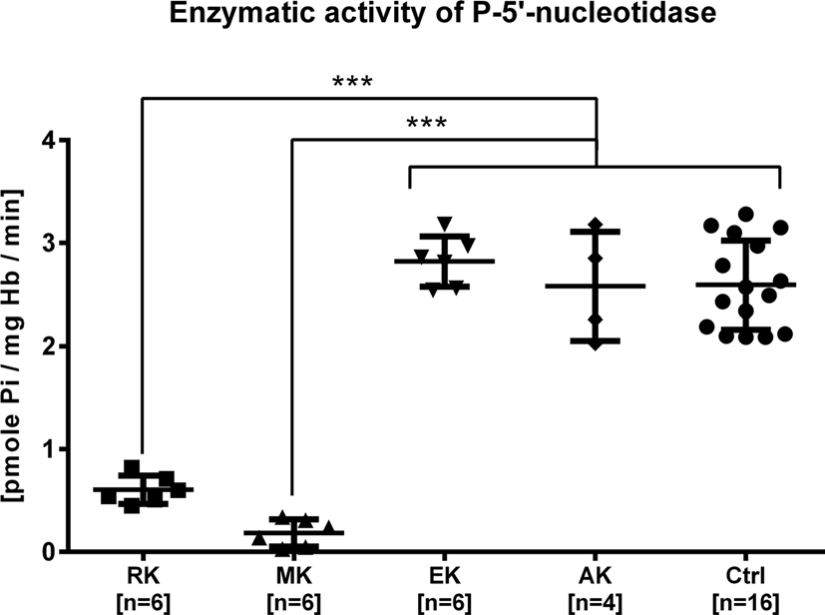
Significant decline in enzymatic activity in HA patients’ (RK and MK) erythrocytes hemolysate caused by lack of cytosolic pyrimidine 5′-nucleotidase. The results for RK and MK are statistically significant at *p* < 0.001 in relation to asymptomatic family members (EK, AK) (for all family members we used technical replicates) and the control group (for four healthy individuals we used biological and technical replicates). Student’s *t*-test, ***p < 0.001. Error bars represent standard deviation

To determine differences in enzymatic activity of cytosolic pyrimidine 5′-nucleotidase between healthy individuals and patients with hemolytic anemia, an enzymatic assay was performed. The inorganic phosphorus liberation during incubation of UMP with hemolysate was measured for four clinically healthy adults (“control group”) as well as for individuals from studied family members. The results depicted in Fig. 4 showed a statistically significant (*p* < 0.05) decrease in pyrimidine 5′-nucleotidase activity in hemolysate of RK and MK patients (fold change 0.21 and 0.07, respectively) in relation to the asymptomatic family members and the control group. In Additional file 1: Fig. S1.5A, the average values from the biological repeats are presented. This result is consistent with the lack of a pyrimidine 5′-nucleotidase band in the erythrocyte cytosol of examined patients, demonstrated by the western blotting technique.

Confirmation of pyrimidine ribonucleotide degradation blockage was obtained by purine/pyrimidine ratio measurement in erythrocytes [46]. The ratio for patients with hemolytic anemia (RK and MK) was approximately 2–2.5 times lower (1.26 ± 0.09) than for the control group (3.17 ± 0.66) and asymptomatic family members (2.88 ± 0.42) (Additional file 1: Fig. S1.5B). The results indicated accumulation of pyrimidine nucleotides in the erythrocyte cytosol in comparison with normal controls and correspond with the above-mentioned pyrimidine 5′-nucleotidase activity results described in the literature.

### Searching for the cause of the presence of only the mutated allele in the transcriptome

**Fig. 5:**
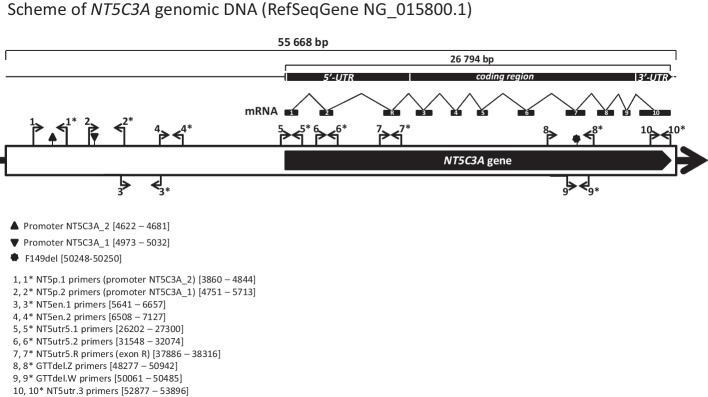
Scheme of *NT5C3A* gene genomic DNA (RefSeqGene NG_015800.1). The amplified gDNA fragments are indicated by arrows

The search for the causes of the presence of only the mutated allele in the transcriptome of the *NT5C3A* gene of both brothers was carried out in a few steps. Verification began with the analysis of the promoter and 5′-UTR region in the *NT5C3A* gene including the known transcription factor binding sites (Fig. 5). The detected polymorphisms did not appear to be significant for 5ʹ-nucleotidase expression (Additional file 1: Table S1.14). The reason for such conclusions was the occurrence of changes in all family members, including the asymptomatic ones, as well as the high frequency of most of them. Furthermore, the results of the molecular karyotype analysis performed with the array-CGH method did not reveal any abnormalities in the genetic material of the patient with hemolytic anemia (RK), or in the asymptomatic father of this patient (AK).

**Fig. 6:**
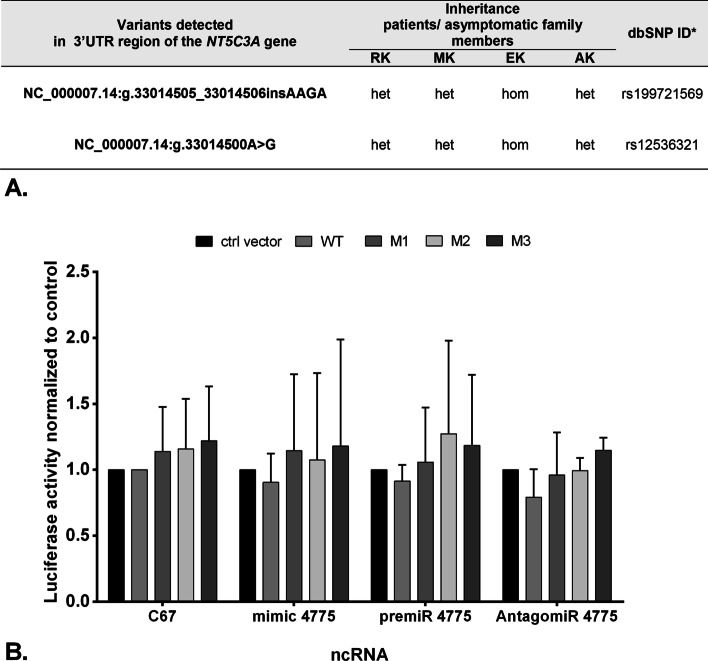
Searching for the cause of the presence of a single allele of the *NT5C3A* gene in the transcriptome. Luciferase reporter assays. **A** Variants present in studied family members located in 3′-UTR of the *NT5C3A* gene identified using the Sanger method. **B** Post-transcriptional activity of the 3′-UTR regions of the human RBC’s specific nucleotidase isoform (cN-IIIA-R) and three different mutation variants tested by transfecting HEK293T cells with the constructs or with control plasmid did not show any significant differences for the phenotype. Constructs used to analysis: human 3′ untranslated region (UTR) of the wild type of the *NT5C3A* gene [WT-HmiT067108-MT06; cN-IIIA-R; transcript variant 4 (NM_001166118.3)] and three different mutation variants (M1 with substitution T/C, M2 insertion TCTT and M3 with both, substitution T/C and insertion TCTT) of firefly luciferase reporter constructs (HmiT018551-MT06-01/02/03, respectively) were purchased from GeneCopoeia (Rockville, MD, USA)

The next possible research hypothesis explaining the presence of only mutated allele in the transcriptome of the *NT5C3A* gene was the appearance of a change in gDNA that would result in abnormal regulation of miRNA expression, in particular miRNAs, which emerged as important regulators of gene expression at the post-transcriptional level in the regulation of different stages of erythropoiesis [56]. Firstly, the data obtained from WES were thoroughly searched for mutant miRNA sequences that could regulate the *NT5C3A* gene expression. This approach did not allow us to identify variants that would seem to be potentially significant. Secondly, we attempted to explain the hypothesis that known miRNAs block expression owing to polymorphisms occurring in the 3′-UTR of the *NT5C3A* gene. We detected two variants in the 3′-UTR region (rs199721569 and rs12536321) (Fig. 6A). We made an attempt to search for an miRNA that could bind to a single or complex hetero- and homozygote. Our hypothesis indicated that a double homozygous would not block expression (as in the case of an asymptomatic sister), while a compound heterozygote (identified in brothers and father) could be negatively regulated by miRNAs. Analysis of miRNAs potentially relevant to the *NT5C3A* gene transcript using the DLR technique showed that in the case of miR-4475 the relationship with the target was not confirmed (Fig. 6B).

Another research approach in searching for the causes of the presence of only the mutated allele in the transcriptome concerned the potential methylation of the *NT5C3A* gene. Recently, studies have shown a different regulatory function for the gene and the potential influence of methylation of this gene on allergic diseases [57, 58]. The CpG-rich fragment of the *NT5C3A* gene, after the bisulfite conversion of cytosines, showed no methylated DNA for all samples. Primers used for unconverted templates (NT5C3A_gDNA) showed no amplification product bisulfite sequencing PCR. All amplicons for gDNA were produced. Additional file 1: Fig. S1.2B shows a fragment of the chromatogram obtained for BSP for one of the brothers (RK). The sequence was converted for all cytosines, including the CpG island regions. The CpG-rich fragment, after the bisulfite conversion of unmethylated cytosine show an absence of 5-methylcytosine. In this case, the results also exclude any correlation of such a methylation disturbance with the phenotype.

Details of above-mentioned experimental protocols are provided in Additional files (Additional file 1: Table S1.5, Figs. S1.1 and S1.2). None of the three analyses described above allowed us to discover the reasons for the expression of only one allele of the *NT5C3A* gene. The research should be continued.

## Discussion

Advances in molecular technologies, in particular next-generation sequencing, inspire us to apply these technologies as a first-line approach for the identification of potential mutations and to determine novel causative genes in patients showing a rare form of hemolytic anemia. By using several highly efficient methods of genome, transcriptome, and functional analysis, we have been able to initially characterize the possible nature of the molecular basis of two cases of very rare hemolytic anemia. Preliminary analysis of the gene expression level (RNA-seq) did not show statistically significant differences compared with the controls (healthy subjects and patients with HS).

Using the whole-exome sequencing method allowed us to identify several genome sequence changes that may be a direct cause for the clinical manifestation observed in both patients. Analyses did not identify variants causing the studied case of hemolytic anemia that were present in the Human Gene Mutation Database (HGMD). On the basis of the results of several-stage verification (Additional file 1: Fig. S1.3), which in our opinion indicate the compound nature of the molecular basis of the HA phenotype observed in the studied family, we attempted to identify variants that are part of the common metabolic pathway. Using the GeneAnalytics tool allowed us to select several genes probably relevant for the HA phenotype. The results of the biological pathway analysis presented in Table 3 indicate that the genes associated with the highest score are involved in the purine and pyrimidine metabolism. One possible cause of the disease may be mutations in the four genes coding for the proteins (*NT5C3A*, *TYMP*, *SCO2*, and *PUDP*). Known functions as well as pathways of normal and pathological gene products of these genes are presented in Table 3. Among the changes selected in this way was the variant located in the *NT5C3A* gene described above (rs1227859962). Mutations detected in the *TYMP* and *SCO2* genes (rs11479, rs112723255) and *PUDP* (rs187333600), although not yet correlated with the HA phenotype, may also cause additional accumulation of pyrimidine metabolites in the erythrocytes of the studied family. The aforementioned 5′-nucleotidase encoded by the *NT5C* gene dephosphorylates dUMP, which in turn is a phosphorylase substrate encoded by the *TYMP* gene [59]. The STRING database predicts functional interactions between proteins encoded by *TYMP* and *SCO2* genes. Two changes identified in both brothers (located at a short distance from each other) detected within the coding sequence for the *TYMP* gene (missense mutations) simultaneously include the promoter sequence of the *SCO2* gene. Both mutations constitute a compound heterozygote, and each of the changes is inherited from another parent. The STRING database predicts functional interactions between proteins encoded by *TYMP* and *NT5C3A* genes (ENSP00000242210, score 0.9) as well as several other genes encoding 5′-nucleotidases (*NT5C1A*, *NT5C*, *NT5C2*, *NT5M*).

An in-depth analysis of the variant and gene data summarized in Tables 2 and 3 led us to conclude that the variant detected in the *NT5C3A* gene appears to be the key phenotype. In the nucleotide sequence of genes associated with known erythrocyte pathologies, only one variant was found to be very rare and at the same time potentially significant. The deletion of phenylalanine localized to two amino acid residues from the substrate binding site for this enzyme [60] has not been described in the literature before. The p.F149del mutation (NP_001159590.1, rs1227859962) in the *NT5C3A* gene has been detected only in the patients in the heterozygous inheritance (RK, MK) and is absent in the sister and father (EK, AK). This rare mutation has been described so far in one Estonian case (1/4480, Estonian; 1/31406, GnomAD). The authors inform us that this deletion was detected in a heterozygous state in one individual in the Estonian Genome Centre database (EGC), and when sequencing was performed this person was categorized as a healthy individual. As mentioned above, mutations in the *NT5C3A* gene are the cause of nonspherocytic hemolytic anemia associated with pyrimidine 5′-nucleotidase deficiency, which was observed only in the case of recessive inheritance or in double heterozygotes (OMIM #266120, #606224). The pattern of inheritance of this mutation in our patients excludes the possibility that only one mutation is responsible for the clinical symptoms. This allows us to propose a hypothesis indicating the compound nature of the potential causes of the HA phenotype in the studied family.

The expression of the *NT5C3A* gene falls, as evidenced by the presence of the gene transcript in the early (CD71^++++^) reticulocyte maturation stages and the absence in the late stage (GPA^++^) (Additional file 1: Table S1.9). This may indicate a similar mechanism of decrease in the activity of this enzyme during the erythrocyte life span. Analysis of changes identified in the studied family by WES in genes coding for known mammalian 5′-nucleotidases (Additional file 1: Table S1.15) with their biological function (Additional file 1: Table S1.16) did not reveal another change (apart from p.F149del) directly affecting the phenotype.

Functional analysis of the enzymatic activity of pathological variants of pyrimidine 5′-nucleotidase (cN-IIIA) obtained by site-directed mutagenesis showed a reduction of catalytic efficiency and/or alterations of thermal stability [13, 16, 28]. All mutations investigated are expected to lead to decay of intracellular pyrimidine 5′-nucleotidase activity. In contrast to D87V and N179S of patients exhibiting only residual (2–5%) 5′-nucleotidase activity, in the case of the other variants (L131P, G230R, and N179S) much higher activity was demonstrated despite substantial changes in the kinetics and thermostability parameters of mutant enzymes [16]. As some authors pointed out, in some cases, pyrimidine 5′-nucleotidase deficiency could be compensated, possibly by other nucleotidases and/or by alternative pathways in nucleotide metabolism, preventing estimation of the true activity of pyrimidine 5′-nucleotidase [15, 16]. On the other hand, deficiency of pyrimidine 5′-nucleotidase can also be observed in the case of other diseases (thalassemia or certain lymphomas) and as a consequence of lead poisoning [12, 61, 62].

As shown by studies on other diseases, single-amino-acid-deletion mutants resulted in the complete loss of function [63–65]. The phenotypic effects of such deletions of course also depend on the location of the amino acid residue and its effect on the protein structure. In the case of the discussed mutation, its localization and potential influence on interactions seem to be highly significant. From the crystal structure of human cytosolic 5′-nucleotidase III (NT5C3) (PDBe;2cn1) [60], we know that the substrate binding site (NM_001166118.3:Ser152Ala153) and the phenylalanine crucial for both affected patients (NM_001166118.3:Phe149) are located in the same β4 strand of the reticulocyte 5′-nucleotidase (NM_001166118.3: 199-155aa: VFIFSAGI; Additional file 1: Fig. S1.6).The p.F149del causes shortening of the beta strand (mut:VIFSAGI), which most likely destabilizes the enzyme structure, preventing binding of the substrate. The biological function of the enzyme is lost, resulting in the near complete lack of the enzyme’s activity observed in the brothers. The mutation can also cause a gross conformational change that makes the enzyme molecule unstable.

The heterozygous NM_001166118.3 (the reticulocyte mRNA splice variant cN-IIIA-R): c.444_446delGTT mutation in the *NT5C3A* gene encoding cytosolic pyrimidine 5′-nucleotidase (p.F149del), most likely inherited from the mother, is the only copy expressed in patients. The correct allele consistent with the reference sequence was not detected in the cDNA of both brothers. Reticulocyte transcriptome analysis showed decreased 5′-nucleotidase gene expression levels in patient MK. This result correlates with the absence of cytosolic pyrimidine 5′-nucleotidase in both brothers and protein deficiency in the father. The molecular basis of this state remains unsolved. It is most likely the result of the presence of only a mutated allele in the transcriptome of the *NT5C3A* gene in affected patients and probably also the single allele in the father, regulated via an unknown mutation. The main reasons for this hypothesis are: (1) heterozygous mutation in patients results in almost complete loss of enzyme activity; (2) heterozygosity is not observed at the mRNA/cDNA level (Fig. 2B and Additional file 1: Fig. S1.4.B); (3) the mutation results in the absence of 5′-nucleotidase in WB (Fig. 3B, D); (4) the level of the 5′-nucleotidase protein in the father’s erythrocytes is lower than in his asymptomatic daughter, but the enzyme activity is near normal (Figs. 3 and 4). Possible reasons for inhibiting expression from one allele may be a mutation related to another miRNA [66], or a mutation affecting the regulatory factors responsible for the expression of this gene. In the father’s case, however, the correct allele is expressed, but the expression of only a single allele is responsible for the decreased protein level. The lower expression level is still high enough to ensure the activity of the enzyme in the father’s erythrocytes. The clinical manifestations of the disease are, as mentioned previously, observed only in the case of recessive inheritance or compound heterozygotes. In the case of both brothers, the expression of only the mutated, nonfunctional 5′-nucleotidase leads to a dramatic decrease/lack of enzyme activity in their erythrocytes. The effect of decreasing enzyme activity is the accumulation of pyrimidines in the cells, which was proved by the reduction of the purine/pyrimidine ratio. The 2.5-fold reduction found in the brothers correlates with the literature data. While there is a possibility that there is one or more spontaneous mutations, it is highly unlikely. Without the mother’s genetic material, it is impossible to verify this possibility. The results of functional tests presented above confirm the correlation of the molecular analysis results with the phenotype of the pyrimidine 5′-nucleotidase deficiency.

## Conclusions

Molecular studies performed by WES analysis in the HA family reported here permit the conclusion that both affected probands have a compound form of the defect. By applying the two highly efficient methods of genome and transcriptome analysis, we were able to initially characterize the apparent nature of the molecular basis of these unique cases of hemolytic anemia, not previously described. We detected a very rare mutation, a heterozygous deletion in the *NT5C3A* gene (c.444_446delGTT), resulting in a single amino acid residue deletion (p.F149del) in cytosolic pyrimidine 5′-nucleotidase in two brothers of Polish origin. Transcriptome analysis of this gene showed the presence of only mutated allele expression and a lack of the presence of nucleotidase protein in the erythrocyte cytosol of both studied patients. Dramatic decreased enzymatic activities of pyrimidine-5′-nucleotidase in the erythrocytes of both brothers, as well as the 2.5-fold reduction of the purine/pyrimidine ratio observed only in the brothers’ erythrocytes, confirms the correlation of the molecular analysis results with the phenotype of the pyrimidine 5′-nucleotidase deficiency. Altogether our results may substantiate the hypothesis that the molecular defect is heterogeneous, involving expression of the mutant allele (c.444_446delGTT). Further studies should be carried out to fully explain the mechanism underlying these rare cases of erythrocyte enzymopathy. First of all, the molecular background of the single allele expression should be studied. These studies may open up a new area concerning regulation of gene expression.

## Supplementary Information

**Additional file 1: **Supplementary figures and tables. This file contains supplementary materials: **Figure S1.1** Constructs used to analysis of the post-transcriptional activity of the 3'-UTR regions of the human RBC’s specific nucleotidase isoform. **Figure S1.2 **A. Primer sequences for bisulfite sequencing PCR (BSP) used for detecting converted templates. B. Sequencing chromatogram for patient RK. Bisulfite converted DNA showing full conversion C → T in the CpG-rich fragment of the NT5C3A gene. The absence of a C-peak indicates the absence of 5-methylcytosine (5mC). **Figure S1.3** Analysis of the workflow applied to prioritize variants found in the whole-exome sequencing (WES) data from the probands. **Figure S1.4** The rare *NT5C3A* gene mutation (rs1227859962) is associated with pyrimidine-5′-nucleotidase deficiency. Fragment of sequencing traces of the *NT5C3A* gene in an affected patient (RK and MK) and asymptomatic family member (EK and AK). **Figure S1.5** Functional analysis. A. Significant decline in enzymatic activity in HA patients’ erythrocyte hemolysate (erythrocytes) caused by lack of cytosolic pyrimidine 5ʹ-nucleotidase. B. Purine/pyrimidine ratio measurement in erythrocytes. HA patients demonstrated 2–2.5 times lower (1.26 ± 0.09) ratio than the control (Ctrl) group (3.04 ± 0.55). **Figure S1.6** According to the AlphaFold Protein Structure Database (AF-A0A090N7U2-F1) the substrate binding site (marked in red) (NM_001166118.3:Ser152Ala153) and the phenyl-alanine crucial for both HA patients (NM_001166118.3:Phe149; marked in pink) are located in the β4 strand of the reticulocyte 5ʹ-nucleotidase (NM_001166118.3: 199-155aa; VFIFSAGI). **Table S1.1** Blood test results of the studied HA patient (RK). **Table S1.2** PCR primer sequences used for *NT5C3A* genotyping. **Table S1.3** PCR primer sequences used for amplification of the transcript variant 4 of the *NT5C3A* gene. **Table S1.4** Primer sequences for bisulfite sequencing PCR (BSP) and control PCR (primers for gDNA) used for detecting converted/unconverted templates. Both primer pairs were homologous to sequences with a similar chromosomal location. **Table S1.5** Complete blood count (CBC) results of the studied family members. **Table S1.6** Quality control of WES raw reads. **Table S1.7** Summary of variants (mutations/polymorphisms) types of translation impact for all variants identified in both patients with HA. **Table S1.8** Polymorphisms/mutations present in both brothers involved in RBC pathologies identified using the WES analysis of studied family members. **Table S1.9** Comparison between quantity of mutated genes (mutations/polymorphisms present in both brothers) obtained from Exome-Seq analysis, and respective genes deposited in the UniGene NCBI databases: CD71^++++^ library (Lib.8975) and GPA^++^ library (Lib.11923). **Table S1.10** List of variants whose frequency does not exceed 10% identified in the studied family using the WES analysis. **Table S1.11** Number of variants potentially significant for the HA phenotype identified by WES and filtered by *Ingenuity Variant Analysis* tool in the studied family members. **Table S1.12** Selectively enriched biological pathways were identified with gene set enrichment analysis. **Table S1.13** List of variants identified in cDNA using Sanger sequencing. **Table S1.14** Polymorphisms/mutations present in studied family members located in the *NT5C3A* gene identified using the Sanger method. **Table S1.15** Polymorphisms/mutations present in both brothers located in genes encoding known 5′-nucleotidases involved in pyrimidine and purine metabolism identified using the WES analysis of studied family members. **Table S1.16** List of genes encoding known 5′-nucleotidases involved in pyrimidine and purine metabolism and their biological function.

**Additional file 2:** Detailed data on the analyzed variant NM_001166118.3: c.444_446delGTT detected WES and analyzed using *Ingenuity Variant Analysis* plugin (QIAGEN, CA, USA).

**Additional file 3:** Statistically significant differences in gene expression were detected using RNA-Seq for 9 transcripts, whose expression was decreased only for patient RK vs the healthy control (CtrlH). Statistical significance was accepted as a *p*-value of < 0.05 used for most analyzes besides NGS analysis where the FDR corrected *p*-value, *q*-value of < 0.05 was considered as significant.

**Additional file 4:** Supplementary raw WB data.

## Data Availability

All data generated or analyzed during this study are included in this article and its additional files. WES datasets used and/or analyzed during this study are available from the corresponding author upon reasonable request.

## Abbreviations

*AK1*: Gene encoding adenylate kinase 1
AMP: Adenosine monophosphate
*ANK1*: Gene encoding ankyrin 1
*ATF*: Gene encoding apolipoprotein E
*ATF3*: Gene encoding activating transcription factor 3
AZT: Azidothymidine
*BPGM*: Gene encoding bisphosphoglycerate mutase
BSP: Bisulfite sequencing polymerase chain reaction
CADD: Combined annotation-dependent depletion
cDNA: Complementary DNA
CD71: Transferrin receptor 1
*CDK1*: Gene encoding cyclin dependent kinase 1
CGH: Comparative genomic hybridization
*CHEK1*: Checkpoint kinase 1
*CLDN6*: Claudin 6
CMP: Cytidine monophosphate
cN-IIIA-R: Reticulocyte isoform of cytosolic pyrimidine 5′-nucleotidase
dCMP: Deoxycytidine monophosphate
*DNAH11*: Dynein axonemal heavy chain 11
DLR: Dual-luciferase reporter
dTMP: Deoxythymidine monophosphate
dUMP: Deoxyuridine monophosphate
EDTA: Ethylenediaminetetraacetic acid
EMA: Eosin-5-maleimide
*ENO1*: Gene encoding enolase 1
*EPB41*: Gene encoding erythrocyte membrane protein band 4.1
*EPB42*: Gene encoding erythrocyte membrane protein band 4.2
GAPDH: Glyceraldehyde-3-phosphate dehydrogenase
G6PD: Glucose-6-phosphate dehydrogenase
*GCLC*: Gene encoding glutamate-cysteine ligase catalytic subunit
gDNA: Genomic deoxyribonucleic acid
*GEMIN8*: Gene encoding gem nuclear organelle associated protein 8
GPA: Glycoprotein A
*GPI*: Gene encoding glucose-6-phosphate isomerase
*GPX1*: Gene encoding glutathione peroxidase 1
*GSR*: Gene encoding glutathione-disulfide reductase
*GSS*: Gene encoding glutathione synthetase
HA: Hemolytic anemia
Hb: Hemoglobin
*HBA1*: Gene encoding hemoglobin subunit alpha 1
*HBA2*: Gene encoding hemoglobin subunit alpha 2
*HBB*: Gene encoding hemoglobin subunit beta
*HCFC1*: Gene encoding host cell factor C1
HCT: Hematocrit
HEK293T: 293T cell line human
HGMD: Human Gene Mutation Database
HHA: Hereditary hemolytic anemia
*HK1*: Gene encoding hexokinase 1
HRP: Horseradish peroxidase
HS: Hereditary spherocytosis
IVA: Ingenuity Variant Analysis
KEEG: Kyoto Encyclopedia of Genes and Genomes
*KCNN4*: Gene encoding potassium calcium-activated channel subfamily N member 4
m7GMP: 7-Methyl-guanosine-5′-monophosphate
MEST: Mesoderm-specific transcript
MFI: Mean fluorescence intensity
miRNA: MicroRNA
mRNA: Messenger RNA
NADPH: Reduced nicotinamide adenine dinucleotide phosphate
NCBI: National Center for Biotechnology Information
ND: Not determined
*NGFRAP1*: Gene encoding nerve growth factor receptor (TNFRSF16) associated protein 1 (NGFRAP1) pseudogene
NGS: Next-generation sequencing
*NOP56*: Gene encoding NOP56 ribonucleoprotein
*NT5C*: Gene encoding 5′, 3′-nucleotidase, cytosolic (cN-IIIB)
*NT5C1A*: Gene encoding 5′-nucleotidase, cytosolic IA
*NT5C2*: Gene encoding 5′-nucleotidase, cytosolic II
*NT5C3A*: Gene encoding 5′-nucleotidase, cytosolic IIIA (cN-IIIA; P5N)
*NT5M*: Gene encoding 5′, 3′-nucleotidase, mitochondrial
OMIM: Online Mendelian Inheritance in Man
P5N-I: Type-I isoform of pyrimidine 5′-nucleotidase (297 aa)
P5ND: Pyrimidine 5′-nucleotidase deficiency
p36: Isoform of pyrimidine 5′-nucleotidase identical to p36
PCR: Polymerase chain reaction
*PGK1*: Gene encoding phosphoglycerate kinase 1
*PIEZO1*: Gene encoding piezo type mechanosensitive ion channel component 1
*PKLR*: Gene encoding pyruvate kinase L/R
PM1: Pathogenic moderate 1
PM2: Pathogenic moderate 2
PM4: Pathogenic moderate 4
POLE: Genomic encoding DNA polymerase epsilon, catalytic subunit A
PUDP: Gene encoding pseudouridine 5′-phosphatase
qPCR: Real-time polymerase chain reaction
*RAD51C*: Gene encoding RAD51 paralog C
RBC: Red blood cell
Ret: Reticulocytes
*RHAG*: Gene encoding Rh-associated glycoprotein
RNA-seq: RNA sequencing
RNU44: Small nucleolar RNA, C/D Box 44
*SCO2*: Gene encoding synthesis of cytochrome C oxidase 2
SDS–PAGE: Sodium dodecyl sulfate–polyacrylamide gel electrophoresis
*SLC4A1*: Gene encoding solute carrier family 4 member 1 (Diego Blood Group)
*SPTA1*: Gene encoding spectrin alpha, erythrocytic 1
*SPTB*: Gene encoding spectrin beta, erythrocytic
STRING: Search Tool for the Retrieval of Interacting Genes/Proteins
TBS: Tris-buffered saline
TBS-T: Tris-buffered saline with Tween 20
*TPI1*: Gene encoding triosephosphate isomerase 1
*TYMP*: Gene encoding thymidine phosphorylase
*UBE2E1*: Gene encoding ubiquitin conjugating enzyme E2 E1
UMP: Uridine monophosphate
UMPH-1: Uridine-5-monophosphate hydrolase-1, official name NT5C3A, encoded by *NT5C3A* gene
3′-UTR: 3′-Untranslated region
WB: Western blot
WES: Whole-exome sequencing
*XK*: Gene encoding X-linked Kx blood group antigen, Kell and VPS13A binding protein
*ZDHHC17*: Gene encoding zinc finger DHHC-type palmitoyltransferase 17
