# Supplementary material for: A rare mutation (p.F149del) of the NT5C3A gene is associated with pyrimidine 5′-nucleotidase deficiency
Source: Cell Mol Biol Lett. 2022 Nov 24;27:104. doi: 10.1186/s11658-022-00405-w (PMC9700897; doi:10.1186/s11658-022-00405-w)
Supplement: Supplementary file 1 — Additional file 1: Supplementary figures and tables. This file contains supplementary materials: Figure S1.1 Constructs used to analysis of the post-transcriptional activity of the 3'-UTR regions of the human RBC’s specific nucleotidase isoform. Figure S1.2 A. Primer sequences for bisulfite sequencing PCR (BSP) used for detecting converted templates. B. Sequencing chromatogram for patient RK. Bisulfite converted DNA showing full conversion C → T in the CpG-rich fragment of the NT5C3A gene. The absence of a C-peak indicates the absence of 5-methylcytosine (5mC). Figure S1.3 Analysis of the workflow applied to prioritize variants found in the whole-exome sequencing (WES) data from the probands. Figure S1.4 The rare NT5C3A gene mutation (rs1227859962) is associated with pyrimidine-5′-nucleotidase deficiency. Fragment of sequencing traces of the NT5C3A gene in an affected patient (RK and MK) and asymptomatic family member (EK and AK). Figure S1.5 Functional analysis. A. Significant decline in enzymatic activity in HA patients’ erythrocyte hemolysate (erythrocytes) caused by lack of cytosolic pyrimidine 5ʹ-nucleotidase. B. Purine/pyrimidine ratio measurement in erythrocytes. HA patients demonstrated 2–2.5 times lower (1.26 ± 0.09) ratio than the control (Ctrl) group (3.04 ± 0.55). Figure S1.6 According to the AlphaFold Protein Structure Database (AF-A0A090N7U2-F1) the substrate binding site (marked in red) (NM_001166118.3:Ser152Ala153) and the phenyl-alanine crucial for both HA patients (NM_001166118.3:Phe149; marked in pink) are located in the β4 strand of the reticulocyte 5ʹ-nucleotidase (NM_001166118.3: 199-155aa; VFIFSAGI). Table S1.1 Blood test results of the studied HA patient (RK). Table S1.2 PCR primer sequences used for NT5C3A genotyping. Table S1.3 PCR primer sequences used for amplification of the transcript variant 4 of the NT5C3A gene. Table S1.4 Primer sequences for bisulfite sequencing PCR (BSP) and control PCR (primers for gDNA) used for detecting convert [file 11658_2022_405_MOESM1_ESM.docx]

**Additional file 1 of Bogusławska et al submitted to CMBL**

**WT - transcript variant 4 (NM_001166118.3) HmiT067108-MT06 640 bp (GeneCopoeia, Inc.)**

acaagcattctccaagaagacctctctcctgtgggtgcaattgaactgttcatccgttcatcttgctgagagacttatttataatatatccttactctcgaagtgttccctttgtataactgaagtattttcagatatggtgaatgcattgactggaagctccttttctccacctctctcaacacactcctcaccgtatcttttaacccatttaaaaaaaaaaaaaagc**t**aaaattagaaaaataactccctacttttccaaagtgaattttgtagtttaatgttatcatgcagcttttgaggagtcttttacactgggaaagtttgtagaaattttaaaataagttttatgaaatggtgaaataatatgcatgattttaagtattgccatttttgtaatttgggttattatgctgatggtatcaccatctcttgaaattgtgttaggtttggttattttgtctggggaaaaaatatttactggaaaagactagcagttagtgttggaaaaacctggtggtgtttacaatgttgctaatcattacaaaacattctatattgaagcactgataataaatatgaaatgcaaaacctttttaattctatggtcaaaactaaaaaaaaaaaaaaaaaaaaaaa

**MUTATION VARIANTS:**

**M1 - 217CS-HmiT067108-MT06-01**

>HmiT067108 3'UTR **with substT/C**

acaagcattctccaagaagacctctctcctgtgggtgcaattgaactgttcatccgttcatcttgctgagagacttatttataatatatccttactctcgaagtgttccctttgtataactgaagtattttcagatatggtgaatgcattgactggaagctccttttctccacctctctcaacacactcctcaccgtatcttttaacccatttaaaaaaaaaaaaaagc**C**aaaattagaaaaataactccctacttttccaaagtgaattttgtagtttaatgttatcatgcagcttttgaggagtcttttacactgggaaagtttgtagaaattttaaaataagttttatgaaatggtgaaataatatgcatgattttaagtattgccatttttgtaatttgggttattatgctgatggtatcaccatctcttgaaattgtgttaggtttggttattttgtctggggaaaaaatatttactggaaaagactagcagttagtgttggaaaaacctggtggtgtttacaatgttgctaatcattacaaaacattctatattgaagcactgataataaatatgaaatgcaaaacctttttaattctatggtcaaaactaaaaaaaaaaaaaaaaaaaaaaa

**M2 - 217CS-HmiT067108-MT06-02**

>HmiT067108 3'UTR **with insTCTT**

acaagcattctccaagaagacctctctcctgtgggtgcaattgaactgttcatccgttcatcttgctgagagacttatttataatatatccttactctcgaagtgttccctttgtataactgaagtattttcagatatggtgaatgcattgactggaagctccttttctccacctctctcaacacactcctcaccgtatcttttaacccatttaaaaaaaaaaa**TCTT**aaagctaaaattagaaaaataactccctacttttccaaagtgaattttgtagtttaatgttatcatgcagcttttgaggagtcttttacactgggaaagtttgtagaaattttaaaataagttttatgaaatggtgaaataatatgcatgattttaagtattgccatttttgtaatttgggttattatgctgatggtatcaccatctcttgaaattgtgttaggtttggttattttgtctggggaaaaaatatttactggaaaagactagcagttagtgttggaaaaacctggtggtgtttacaatgttgctaatcattacaaaacattctatattgaagcactgataataaatatgaaatgcaaaacctttttaattctatggtcaaaactaaaaaaaaaaaaaaaaaaaaaaa

**M3 - 217CS-HmiT067108-MT06-03**

>HmiT067108 3'UTR **with substT/C+insTCTT**

acaagcattctccaagaagacctctctcctgtgggtgcaattgaactgttcatccgttcatcttgctgagagacttatttataatatatccttactctcgaagtgttccctttgtataactgaagtattttcagatatggtgaatgcattgactggaagctccttttctccacctctctcaacacactcctcaccgtatcttttaacccatttaaaaaaaaaaa**TCTT**aaagc**C**aaaattagaaaaataactccctacttttccaaagtgaattttgtagtttaatgttatcatgcagcttttgaggagtcttttacactgggaaagtttgtagaaattttaaaataagttttatgaaatggtgaaataatatgcatgattttaagtattgccatttttgtaatttgggttattatgctgatggtatcaccatctcttgaaattgtgttaggtttggttattttgtctggggaaaaaatatttactggaaaagactagcagttagtgttggaaaaacctggtggtgtttacaatgttgctaatcattacaaaacattctatattgaagcactgataataaatatgaaatgcaaaacctttttaattctatggtcaaaactaaaaaaaaaaaaaaaaaaaaaaa

**Figure S1.1 Constructs used to analysis of the post-transcriptional activity of the 3'-UTR regions of the human RBC’s specific nucleotidase isoform.** Human 3′ untranslated region (UTR) of the wild type of the *NT5C3A* gene (WT -HmiT067108-MT06) and 3 different mutation variants (M1 with substitution T/C, M2 insertion TCTT and M3 with both, substitution T/C and insertion TCTT) firefly luciferase reporter constructs (HmiT018551-MT06-01/02/03, respectively) were purchased from GeneCopoeia, Inc. (Rockville, MD, USA).

A. **NT5C3A_bsDNA Forward primer**

gDNA 5’-CTA**TCCCTTGGAAGACACTAGGCAGGAGAG**AAC-3’

:||**|:::||||||||:|:||||:|||||||**||:

bsDNA 5’-TTA**TTTTTTGGAAGATATTAGGTAGGAGAG**AAT-3’

**>>>>>>>>>>>>>>>>>>>>>>>>>>>**

**NT5C3A_bsDNA Reverse primer**

gDNA 5’-CTC**GTGAGACCCTGAGTCTGCTGAAGGC**CGC-3’

:|+**+|||||:::|||||:||:||||||:**++:

bsDNA 5’-TTC**GTGAGATTTTGAGTTTGTTGAAGGT**CGT-3’

**<<<<<<<<<<<<<<<<<<<<<<<<<**

3’-**CACTCTAAAACTCAAACAACTTCCA**-3’

**B. patient RK**


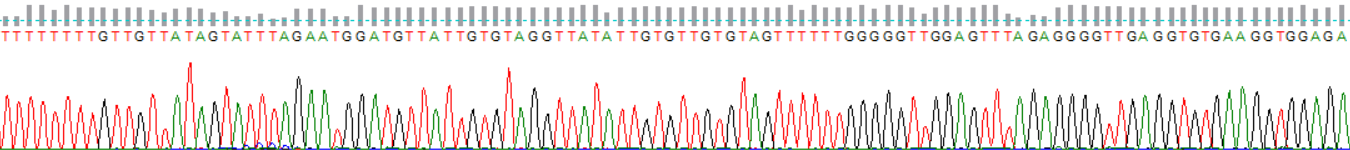


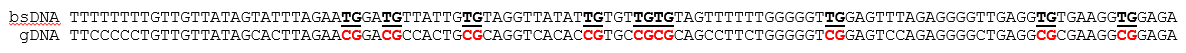


**Figure S1.2** **A.** Primer sequences for bisulfite sequencing PCR (BSP) used for detecting converted templates. **B.** Sequencing chromatogram for patient RK. Bisulfite converted DNA showing full conversion C→T in the CpG-rich fragment of the *NT5C3A* gene. The absence of a C-peak indicates the absence of 5-methylcytosine (5mC).

**Patients with hemolytic anemia (HA)** (2 patients)

**Variants common for HA patients**
27460 variants identified

**Variants deposited in the HGMD**

365 variants identified

**Functional filtering – nonsense, frame shift, splice site, missense (Table S1.6)**
2523 variants identified

**Genes (14) involved in known RBC pathologies (Table S1.7)**
68 variants identified

**Variants associated with autosomal recessive or sex-linked inheritance**
544 variants identified

**Variants with low frequency ≤0.5%**

53 variants identified

**Variants with low frequency ≤0.5%** **according to the 1000Genomes / ExAC datasets**

15 variants identified

**Genes expressed in CD71^++++^ and GPA^++^ type cells (Table S1.8)**
842 variants identified

**Variant Filtering and Interpretation Using Ingenuity Variant Analysis (IVA) (Table S1.10)**

**Common Variants**  429 variants identified

**Predicted Deleterious** 98 variants identified

**Genetic Analysis** 16 variants identified

**Biological Context**  3 variants identified

**Variants with frequency ≤10% that associated with a recessive pattern of inheritance, potential compound heterozygotes, and additional genes correlated with the anemia phenotype (Table S1.9)**

186 variants identified

- ***GeneAnalytics^TM^* tools /Pathways/Biological Process (Table S1.11)**

7 potentially important variants identified

**Variants potentially important for HA phenotype (Tables 2 and 3)**
9 variants identified

- **Proteins responsible for purine and pyrimidine metabolism**
   4 variants were identified in 4 genes (***NT5C3A, TYMP, SCO2, PUDP)***
- COMPOUND HETEROZYGOTES pattern of inheritance

**Heterozygous mutation (p.F149del) and a single mutant allele expression
are associated with pyrimidine-5'-nucleotidase deficiency**

**Figure S1.3** Analysis of the workflow applied to prioritize variants found in the whole-exome sequencing (WES) data from the probands. The summary contains only variants common for HA patients.

**A**.
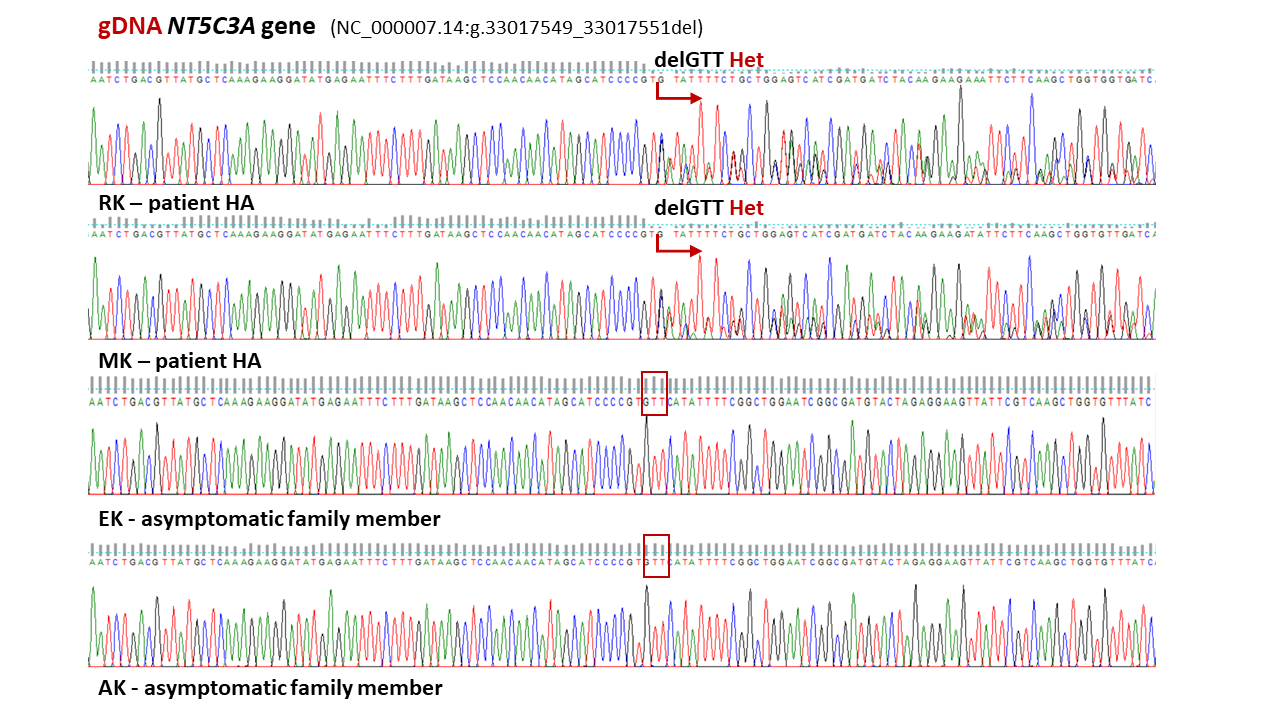


**B**.
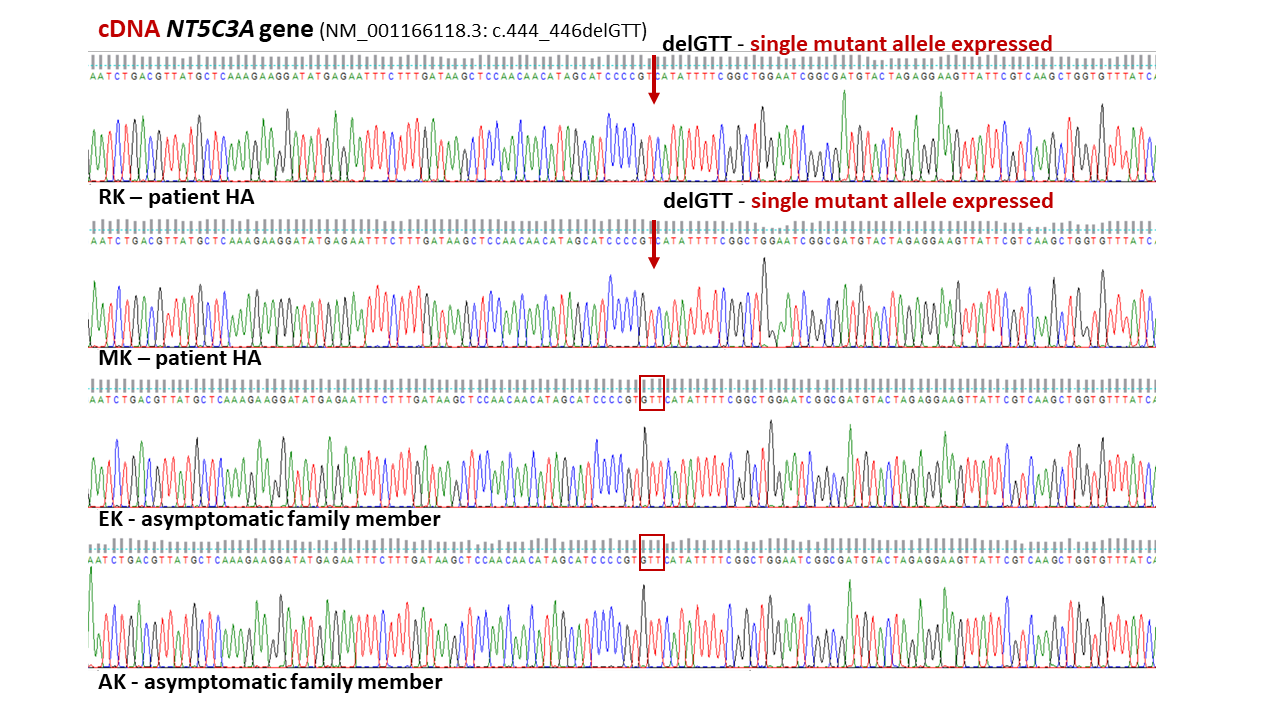


**Figure** **S1.4** The rare *NT5C3A* gene mutation (rs1227859962) is associated with pyrimidine 5'-nucleotidase deficiency. Fragment of sequencing traces of the *NT5C3A* gene in an affected patient (RK and MK) and asymptomatic family member (EK and AK). The gDNA sequence analysis **A.** revealed a **heterozygous deletion** (NC_000007.14:g.33017549_33017551del) only in both affected patients in contrast to cDNA sequence analysis **B.** revealed **only one mutant allele with deletion** (NM_001166118.3: c.444_446delGTT) causing the single amino acid deletion (NP_001361265.1:p.Phe149del) in cytosolic pyrimidine 5’-nucleotidase.

**A.**
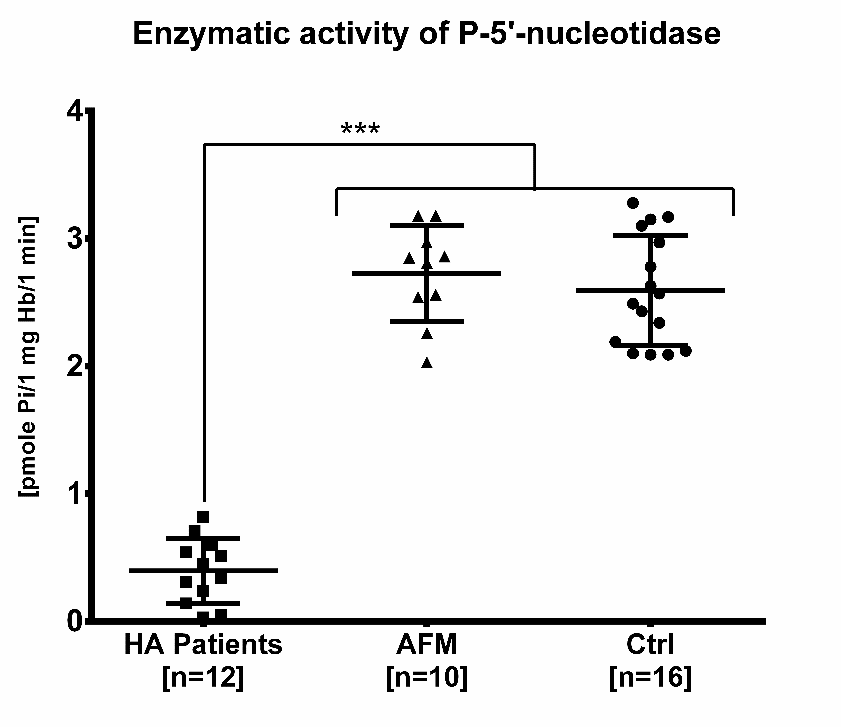
 **B.**



**Figure** **S1.5 Functional analysis. A.** Significant decline in enzymatic activity in HA patients' erythrocyte hemolysate (erythrocytes) caused by lack of cytosolic pyrimidine 5’-nucleotidase. The results for HA patients are statistically significant at p<0.001 in relation to asymptomatic family members (AFM) and the control group (Ctrl). Student T-test, *** - p<0.001. Error bars represent standard deviation. n number corresponds to technical replicates (2 biological samples for HA, 2 for AFM and 4 for Ctrl). **B.** Purine/pyrimidine ratio measurement in erythrocytes. HA patients demonstrated 2 - 2.5 times lower (1.26±0.09) ratio than Ctrl group (3.04±0.55). Data are expressed as relative unit (n - number corresponds to biological replicates). Error bars represent range.


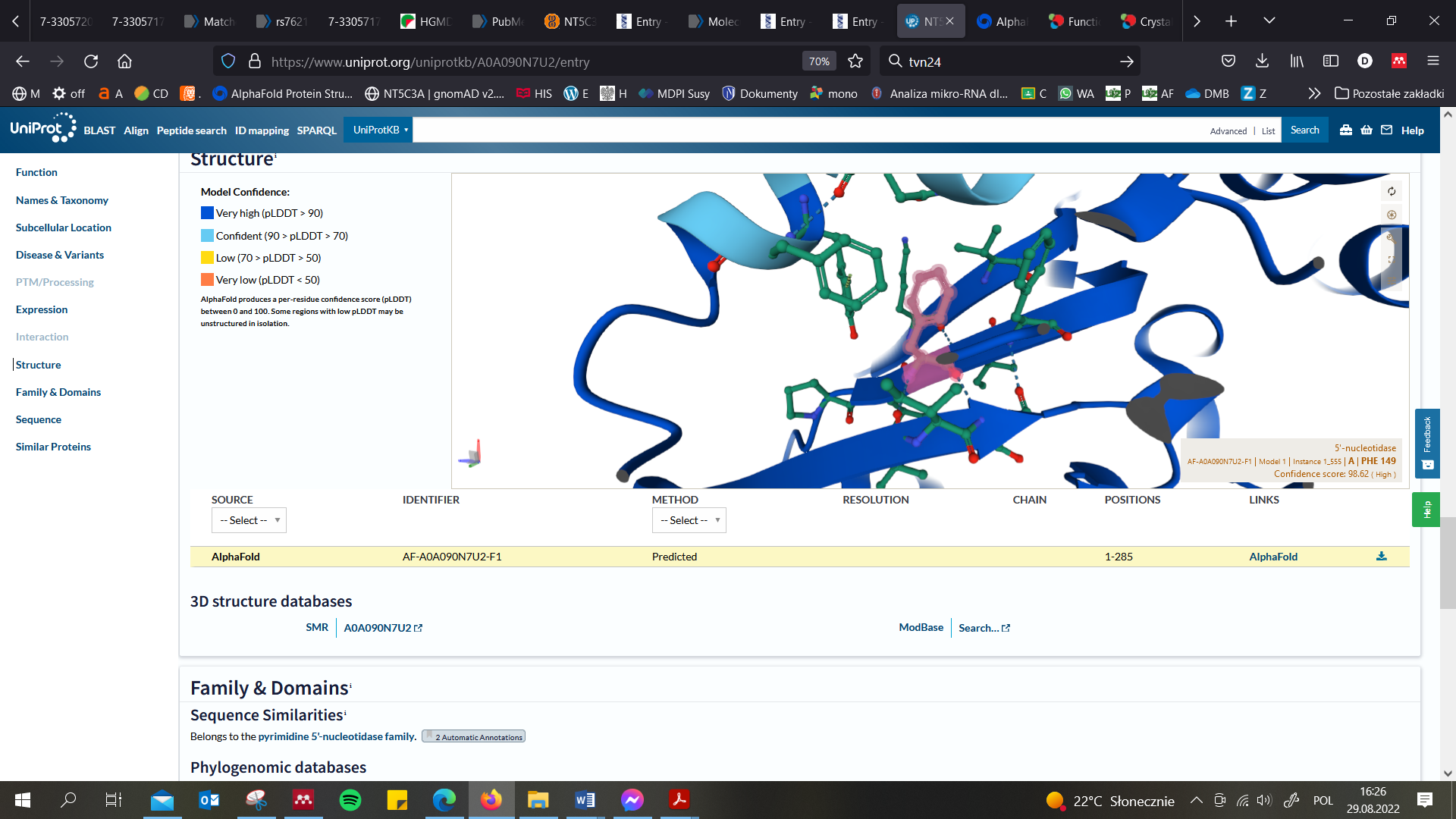


**Substrate binding site**

**Figure** **S1.6** According to AlphaFold Protein Structure Database (AF-A0A090N7U2-F1) the substrate binding site (marked in red) (NM_001166118.3:**Ser152Ala153**) and the crucial for both HA patients phenylalanine (NM_001166118.3:**Phe149**; marked in pink) are located in the β4 strand
of the reticulocyte 5’-nucleotidase (NM_001166118.3: 199-155aa; **V**F**IF**SA**GI).** <https://www.uniprot.org/uniprotkb/A0A090N7U2/entry>

**Table S1.1** Blood test results of the studied HA patient (RK).

| **Test name** | **Result** | **Unit** | **Reference range** | **↑↓** |
| --- | --- | --- | --- | --- |
| Direct antiglobulin test (DAT) | negative |  |  |  |
| Indirect antiglobulin test (IAT) | negative |  |  |  |
| Reticulocytes | 78.9 | per 1000 | 7-20 | **↑** |
| Immature reticulocyte fraction (IRF) | 0.63 | index | 0.2-0.4 | **↑** |
| Mean reticulocyte volume (MRV) | 137.5 | fl | 92-100 | **↑** |
| Lactate dehydrogenase (LDH) | 611 | U/L | <250 | **↑** |
| Rheumatoid factor (RF) | <20 |  | <30 |  |
| Β2 microglobulin (B2M) | 1.66 | mg/L | <2.5 |  |
| Erythropoietin (EPO) | 31.9 | mLU/mL | 5.4-31.0 | **↑** |
| Haptoglobin | 1.0 | mg/dL | 200-300 | **↓** |
| Sodium | 137 | mmol/L | 135-145 |  |
| Potassium | 4.2 | mmol/L | 3.6-5.0 |  |
| Calcium | 2.37 | mmol/L | 2.1-2.6 |  |
| Iron | 72 | mmol/L | 35-168 |  |
| Ferritin | 33.2 | ng/mL | 24-336 |  |
| Transferrin | 269 | mg/dL | 200-400 |  |
| Transferrin saturation | 19 | % | 16-45 |  |
| Alkaline phosphatase | 60 | U/L | 40-130 |  |
| Total bilirubin | 2.00 | mg/dL | 0.1-1.2 | **↑** |
| Direct bilirubin | 0.33 | mg/dL | <0.3 | **↑** |
| Indirect bilirubin | 1.67 | mg/dL | <0.8 | **↑** |
| Gamma-GT | 13 | U/L | <64 |  |
| Aspartate aminotransaminase (ASAT) | 48 | U/L | <50 |  |
| Alanine aminotransaminase (ALAT) | 23 | U/L | <50 |  |
| Holotranscobalamin | 52.1 | pmol/L | >50 |  |
| Methylmalonic acid | 18 | μg/L | 9-32 |  |
| Folic acid | 5.3 | μg/L | 2.3-17.5 |  |
| HbA | 97.4 | % |  |  |
| HbA_2_ | 2.6 | % | <3.2 |  |
| HbF | negative |  |  |  |
| **Erythrocyte enzymes** (μmol substrate-turnover/10^11^ erythrocytes/min.) | | | | |
| Hexokinase | 2.7 |  | 2.3±0.5 |  |
| Glucose phosphate isomerase | 132 |  | 124±13 |  |
| Triosephosphate isomerase | 6927 |  | 6055±705 |  |
| Pyruvate kinase in 0.4x10^-3^ PEP | 28.7 |  | 22.1±7 |  |
| Pyruvate kinase in 2.0x10^-3^ PEP | 52.1 |  | 41±10 |  |
| Glucose-6-phosphate dehydrogenase | 35.3 |  | 30.6±4.5 |  |
| 6-Phosphogluconate dehydrogenase | 26.8 |  | 26.2±4.1 |  |
| Glutathione reductase with NADPH | 29.0 |  | 25.7±3.0 |  |
| Glutamic oxaloacetic transaminase | 20.9 |  | 16.2±5.2 |  |

**Table S1.2** PCR primer sequences used for *NT5C3A* genotyping (NC_000007.14).

| PCR primers | Sequence (5’->3’) | Product size | Description |
| --- | --- | --- | --- |
| NT5p.1S | TGAGACTGTGACCATCTTAGGC | 985 | Distal promoter |
| NT5p.1A | CAGACTCAGGGTCTCACGAGC |  |  |
| NT5p.2S | GAGCGAACCACGGCCTAAT | 963 | Proximal promoter |
| NT5p.2A | TAATCTAACTCACGGCAGCCC |  |  |
| NT5en.1S | ACCGTTGCCGCTAATCTAGT | 1017 | GH07J033060 enhancer - Part 1 |
| NT5en.1A | GGGAGATATGGCAACTCGGT |  |  |
| NT5en.2S | TCCCACATCTGTATGCCCACAGC | 620 | GH07J033060 enhancer - Part 2 |
| NT5en.2A | TGACCAATATGAGCTATGTCTGTTG |  |  |
| NT5utr5.1S | AATCCCACTGCCCTTTGATAAAGAC | 1099 | 5’-UTR – exon 1 |
| NT5utr5.1A | GCTTGTGGCTGTCAGTAAAACT |  |  |
| NT5utr5.2S | CAGTATGCAAAGGACATAGGTTTCA | 527 | 5’-UTR – exon 2 |
| NT5utr5.2A | AGCACGACAATGTGCATTAGATTT |  |  |
| NT5utr5.RS | CTACTGGTTCTCTTTCATCCATTG | 431 | 5’-UTR – exon 3/R (reticulocyte exon) |
| NT5utr5.RA | CCTTAACTGGCAGATGCACAGC |  |  |
| NT5utr.3S | TGCCCAAGAGATCTAACAACGA | 1020 | 3’-UTR |
| NT5utr.3A | GGTCTCAGTATTTTCTGAAGGGCA |  |  |
| GTTdel.zS | TCCAAAATATGCCACCTATCACAA | 2666 | The fragment containing the delGTT (external primers for Nested-PCR) |
| GTTdel.zA | AAGGCTTCAAAGTTGCTGCATTA |  |  |
| GTTdel.wS | CCTCTTGGGCAGTATATTGAAA | 425 | The fragment containing the delGTT (internal primers for Nested-PCR) |
| GTTdel.wA | ACAGTGGGCCTATCGGCTTG |  |  |

**Table S1.3** PCR primer sequences used for amplification of the transcript variant 4 of the *NT5C3A* gene (NM_001166118.3).

| PCR primers | Sequence (5’->3’) | Product size | Description |
| --- | --- | --- | --- |
| NT5_1s | TTCTTTCCAGAGCCAGTTGCC | 362 | Protein coding region |
| NT5_1a | CTGGTTTCTGCTACACATTTTCACA |  |  |
| NT5_2s | TGTGAAAATGTGTAGCAGAAACCA | 998 | Protein coding region |
| NT5_2a | TCAATTGCACCCACAGGAGA |  |  |
| NT5_3s | TGTTGAGCACATTCTGAAAATTGGA | 450 | Protein coding region |
| NT5_3a | AGACTCCTCAAAAGCTGCATGAT |  |  |
| NT5_4s | CTCAACACACTCCTCACCGTA | 363 | Protein coding region |
| NT5_4a | GATTAGCAACATTGTAAACACCACC |  |  |
| NT5v4s | CTTTCCAGAGCCAGTTGCCAG | 365 | 5’-UTR |
| NT5v4a | GAAGGCTGGTTTCTGCTACAC |  |  |
| NT53prA | ACAAGCATTCTCCAAGAAGACC | 572 | 3’-UTR |
| NT53prS | TATCAGTGCTTCAATATAGAATG |  |  |
| NT54.1S | GTGAAAATGTGTAGCAGAAACCAG | 1493 | The fragment containing the delGTT, insTCTT and substitution T>C (external primers for Nested-PCR) |
| NT54.1A | GATTAGCAACATTGTAAACACCACC |  |  |
| NT5_3s | TGTTGAGCACATTCTGAAAATTGGA | 682 | The fragment containing the insTCTT and  substitution T>C (internal primers for Nested-PCR) |
| NT5_4a | GATTAGCAACATTGTAAACACCACC |  |  |

**Table S1.4** Primer sequences for bisulfite sequencing PCR (BSP) and control PCR (primers for gDNA) used for detecting converted/unconverted templates. Both primer pairs were homologous to sequences with a similar chromosomal location.

| **Gene** | **Forward primer** | **Reverse primer** |
| --- | --- | --- |
| NT5C3A_bsDNA | 5'- TTTTTGGAAGATATTAGGTAGGAGAG -3' | 5'- ACCTTCAACAAACTCAAAATCTCAC -3' |
| NT5C3A_gDNA | 5'- CAGCAGACTCAGGGTCTCACGAG -3' | 5'- GCAGGAGAGAACAACTGCAAGCC -3' |

**Table S1.5** Complete blood count (CBC) results of the studied family members.

| **Test name** | **References for male** | **References for female** | **HA patients** | | **Asymptomatic family members** | |
| --- | --- | --- | --- | --- | --- | --- |
|  |  |  | **RK *(male)*** | **MK (male)** | **EK (female)** | **AK (male)** |
| **RBC** *(T/L)* | 4.2-6.0 | 4.0-5.0 | 3.43 | 3.36 | 4.92 | 4.70 |
| **HCT** *[L/L)* | 0.40-0.54 | 0.37-0.47 | 0.34 | 0.30 | 0.43 | 0.44 |
| **Hb** *(mmol/L)* | 8.69-11.17 | 7.54-9.93 | 7.08 | 6.14 | 9.18 | 9.62 |
| **MCH** *(fmol)* | 1.68-2.11 | 1.68-2.11 | 2.11 | 1.80 | 1.86 | 2.05 |
| **MCHC** *(mmol/L)* | 19.83-22.34 | 20.47-22.34 | 21.72 | 19.86 | 21.10 | 21.72 |
| **MCV** *(µm^3^)* | 80-97 | 80-97 | 96 | 91 | 88 | 93 |
| **WBCs** *(G/L)* | 0.004-0.010 | 0.004-0.010 | 0.009 | 0.006 | 0.008 | 0.006 |
| **PLT** *(G/L)* | 0.15-0.45 | 0.15-0.45 | 0.327 | 0.293 | 0.325 | 0.237 |

RBC—erythrocytes; HCT—hematocrit; Hb—hemoglobin; MCH— mean corpuscular hemoglobin;

MCH— mean corpuscular hemoglobin; MCHC— mean corpuscular hemoglobin concentration; MCV— mean corpuscular volume; WBCs — white blood cells; PLT—platelet.

**Table S1.6** Quality control of WES raw reads. Burrows-Wheeler Aligner (BWA) statistics summarize the percentage of uniquely mapped reads, the percentage of properly paired reads, the percentage of duplicated reads, and the average depth for each sample and combined lanes. Samples belonging to series ‘A’ & ‘C’ were isolated from the fresh peripheral blood and samples belonging to series ‘B’ were isolated from the frozen peripheral blood (stored at -80ºC).

|  | **Total # raw reads** | **% mapped to the genome** | **% properly paired** | **% duplication** | **Average depth** | **Lane** | **WES series** |
| --- | --- | --- | --- | --- | --- | --- | --- |
| **RK_1seriesA** | 34 611 097 | 34.558.056 (99.85%) | 34.157.068 (98.72%) | 2.51% | 43.75 | 1 | **Series A** |
| **RK_2seriesA** | 34 531 170 | 34.485.979 (99.87%) | 34.092.134 (98.77%) | 2.50% | 43.66 | 2 |  |
| **MK_1seriesA** | 49 783 154 | 49.703.094 (99.84%) | 49.268.204 (99.00%) | 3.22% | 60.82 | 1 |  |
| **MK_2seriesA** | 49 598 310 | 49.530.458 (99.86%) | 49.107.630 (99.05%) | 3.20% | 60.63 | 2 |  |
| **RK_1seriesB** | 53 083 543 | 53.058.620 (99.95%) | 52.671.400 (99.25%) | 2.07% | 68.75 | 1 | **Series B** |
| **RK_2seriesB** | 54 754 208 | 54.720.345 (99.94%) | 54.292.414 (99.18%) | 2.12% | 70.7 | 2 |  |
| **MK_1seriesB** | 41 163 220 | 41.142.988 (99.95%) | 40.819.438 (99.21%) | 2.88% | 48.23 | 1 |  |
| **MK_2seriesB** | 42 409 252 | 42.382.537 (99.94%) | 42.027.018 (99.14%) | 2.96% | 49.56 | 2 |  |
| **EK_1seriesB** | 70 096 454 | 70.065.013 (99.96%) | 69.574.978 (99.28%) | 5.35% | 89.13 | 1 |  |
| **EK_2seriesB** | 72 152 159 | 72.108.205 (99.94%) | 71.566.060 (99.21%) | 5.50% | 91.4 | 2 |  |
| **AK_1seriesB** | 42 288 224 | 42.271.096 (99.96%) | 41.984.780 (99.32%) | 3.21% | 52.66 | 1 |  |
| **AK_2seriesB** | 43 586 880 | 53.563.242 (99.95%) | 43.247.800 (99.26%) | 3.29% | 54.11 | 2 |  |
| **EK_1seriesC** | 37 889 960 | 37.775.794 (99.70%) | 37.564.516 (99.17%) | 10.10% | 30.72 | 1 | **Series C** |
| **EK_2seriesC** | 36 889 974 | 36.768.246 (99.67%) | 36.524.594 (99.04%) | 9.87% | 29.99 | 2 |  |
| **EK_3seriesC** | 37 927 172 | 37.811.932 (99.70%) | 37.602.704 (99.18%) | 10.12% | 30.79 | 3 |  |
| **EK_4seriesC** | 37 302 223 | 37.182.023 (99.68%) | 36.928.876 (99.03%) | 9.98% | 30.28 | 4 |  |
| **AK_1seriesC** | 34 032 627 | 33.928.658 (99.69%) | 33.749.534 (99.20%) | 10.63% | 29.98 | 1 |  |
| **AK_2seriesC** | 33 155 024 | 33.042.373 (99.66%) | 32.836.546 (99.07%) | 10.42% | 29.28 | 2 |  |
| **AK_3seriesC** | 34 054 174 | 33.949.450 (99.69%) | 33.772.866 (99.20%) | 10.66% | 30.03 | 3 |  |
| **AK_4seriesC** | 33 506 233 | 33.395.266 (99.67%) | 33.182.890 (99.06%) | 10.53% | 29.54 | 4 |  |
| **RK_seriesA/B** | 144 669 685 | 144.591.395 (99.95%) | 143.840.034 (99.43%) | 4.68% | 221.24 | 1,2 | **Combined**  **lines** |
| **MK_seriesA/B** | 141 145 749 | 141.058.999 (99.94%) | 140.394.233 (99.47%) | 6.16% | 212.26 | 1,2 |  |
| **EK_seriesB** | 119 078 500 | 119.033.623 (99.96%) | 118.542.082 (99.56%) | 10.15% | 171.68 | 1,2 |  |
| **AK_seriesB** | 68 822 621 | 68.800.245 (99.97%) | 68.540.381 (99.59%) | 6.19% | 103.58 | 1,2 |  |
| **EK_seriesC** | 112 482 851 | 112.294.772 (99.83%) | 111.879.916 (99.47%) | 28.54% | 98.84 | 1,2,3,4 |  |
| **AK_seriesC** | 109 893 298 | 109.703.582 (99.83%) | 109.296.503 (99.47%) | 28.02% | 97.01 | 1,2,3,4 |  |

**Table S1.7** Summary of variants (mutations/polymorphisms) types of translation impact for all variants identified in both patients with HA.

| **Translation impact of variants** | **Total genes** | | **Genes involved  in known RBC pathologies** | |
| --- | --- | --- | --- | --- |
|  | **Not reported** | **Reported** | **Not reported** | **Reported** |
| Missense mutation | 9 | 2376 | 0 | 6 |
| Frameshift | 0 | 115 | 0 | 2 |
| In-frame | 3 | 127 | 0 | 0 |
| Start lost | 0 | 3 | 0 | 0 |
| Stop gained | 0 | 24 | 0 | 0 |
| Stop lost | 1 | 5 | 0 | 0 |
| **Total variants** | **10** | **2523** | **0** | **8** |

**Table S1.8** Polymorphisms/mutations present in both brothers involved in RBC pathologies identified using the WES analysis of studied family members. The functional variants with potentially large effects are marked with an asterisk. (het – heterozygotic; hom – homozygotic; het-alt - diploid, heterozygous, fully called locus where both alleles differ from the reference)

| **Chr** | **Gene Symbol** | **Position** | **Reference Allele** | **Sample Allele** | **Variation Type** | **Gene Region** | **Protein Variant** | **Genotype** | | | | **dbSNP ID** | **1000 Genomes** | **ExAC** | **HGMD** |
| --- | --- | --- | --- | --- | --- | --- | --- | --- | --- | --- | --- | --- | --- | --- | --- |
|  |  |  |  |  |  |  |  | **RK** | **MK** | **EK** | **AK** |  |  |  |  |
| **RBC membranopathies** | | | | | | | | | | | | | | | |
| 8 | *ANK1* | 41517958 |  | G | Insertion | Intronic |  | Hom | Hom | Hom | Hom | 59908561 | 99,98 | 99,994 |  |
|  |  | 41519248 | T | C | SNV | Intronic |  | Hom | Hom | Hom | Hom | 516946 | 80,431 | 78,53 |  |
|  |  | 41519462 | A | G | SNV | Intronic |  | Hom | Hom | Hom | Hom | 515071 | 78,634 | 78,047 |  |
|  |  | 41529856 | A | G | SNV | Intronic |  | Hom | Hom | Hom | Hom | 508112 | 97,764 | 99,206 |  |
|  |  | 41530482 | C | G | SNV | Intronic |  | Hom | Hom | Hom | Hom | 499295 | 95,228 | 98,794 |  |
|  |  | 41555473 | T | C | SNV | Intronic |  | Hom | Het | Hom | Het | 2241896 | 40,715 |  |  |
|  |  | 158582543 | G | A | SNV | Intronic |  | Het | Het | Het |  | 2251963 | 27,696 |  |  |
|  |  | 158582552 | G | A | SNV | Intronic |  | Het | Het | Het |  | 2251964 | 27,696 |  |  |
|  |  | 158584103 |  | AA | Insertion | Intronic |  | Het | Het | Het |  | 5778083 | 23,922 | 25,098 |  |
|  |  | 158585230 | C | T | SNV | Intronic |  | Het | Het | Het |  | 2518491 | 23,163 | 27,046 |  |
|  |  | 158587390 | C | T | SNV | Intronic |  | Het | Het | Het |  | 857716 | 49,101 | 53,309 |  |
|  |  | 158587784 | T | G | SNV | Intronic |  | Hom | Hom | Hom | Hom | 857717 | 99,98 | 99,986 |  |
|  |  | 158607788 | T | G | SNV | Intronic |  | Hom | Hom | Hom | Hom | 2518492 | 99,98 | 99,981 |  |
|  |  | 158618455 | G | A | SNV | Intronic |  | Het | Het | Het |  | 2246434 | 22,823 | 25,339 |  |
|  |  | 158619728 | A | C | SNV | Exonic | p.S1163A | Hom | Hom | Hom | Hom | 2482965* | 99,461 | 99,826 |  |
|  |  | 158639601 | A | G | SNV | Intronic |  | Het | Het | Het | Hom | 2298801 | 37,72 | 44,137 |  |
|  |  | 158647495 | A | T | SNV | Exonic | p.A314A | Hom | Hom | Hom | Hom | 325996 | 95,687 | 98,497 |  |
|  |  | 158647631 | T | A | SNV | Intronic |  | Hom | Hom | Hom | Hom | 325995 | 95,687 | 98,494 |  |
|  |  | 158647669 | T | C | SNV | Intronic |  | Het | Het | Het |  | 703122 | 45,387 | 54,53 |  |
|  |  | 158655036 | G | A | SNV | Exonic | p.V42V | Hom | Hom | Hom | Hom | 435080 | 96,985 | 99,197 |  |
|  |  | 158656281 | T | C | SNV | Intronic |  | Hom | Hom | Hom | Hom | 2564858 | 96,985 | 99,195 |  |
|  |  | 158656347 |  | AGAG | Insertion | 5'UTR |  | Hom | Hom | Het-Alt | Hom | 111674514 |  | 62,272 |  |
|  |  | 158656432 |  | A | Insertion | 5'UTR |  | Het | Het | Het |  | 59027997 | 40,575 |  |  |

| **Chr** | **Gene Symbol** | **Position** | **Reference Allele** | **Sample Allele** | **Variation Type** | **Gene Region** | **Protein Variant** | **Genotype** | | | | **dbSNP ID** | **1000 Genomes** | **ExAC** | **HGMD** |
| --- | --- | --- | --- | --- | --- | --- | --- | --- | --- | --- | --- | --- | --- | --- | --- |
|  |  |  |  |  |  |  |  | **RK** | **MK** | **EK** | **AK** |  |  |  |  |
| **RBC membranopathies** | | | | | | | | | | | | | | | |
| 16 | *PIEZO1* | 88782676 | A | G | SNV | Exonic | p.P2353P | Hom | Hom | Hom | Hom | 2290902 | 86,781 | 88,956 |  |
|  |  | 88783100 | T | C | SNV | Exonic | p.I2265V | Hom | Hom | Hom | Hom | 1803382* | 19,828 | 29,346 |  |
|  |  | 88783374 | A | G | SNV | Intronic |  | Hom | Hom | Hom | Hom | 8044367 | 89,157 |  |  |
|  |  | 88783521 | T | C | SNV | Exonic | p.P2190P | Hom | Hom | Hom | Hom | 6500491 | 89,197 | 89,648 |  |
|  |  | 88784114 | T | C | SNV | Intronic |  | Hom | Hom | Hom | Hom | 11641326 | 22,664 | 34,094 |  |
|  |  | 88786967 | C | T | SNV | Intronic |  | Hom | Hom | Hom | Hom | 4401037 | 90,076 | 89,203 |  |
|  |  | 88787608 | CTT |  | Deletion | Exonic | p.K1878del | Hom | Hom | Hom | Hom | 150376294* | 21,585 | 35,546 |  |
|  |  | 88787673 | G | A | SNV | Exonic | p.P1857S | Hom | Hom | Hom | Hom | 35159887* | 16,613 | 28,164 |  |
|  |  | 88788903 | T | G | SNV | Intronic |  | Hom | Hom | Hom | Hom | 4238687 | 89,657 |  |  |
|  |  | 88791458 | G | A | SNV | Exonic | p.P1398L | Hom | Hom | Hom | Hom | 11645197* | 17,532 | 32,676 |  |
|  |  | 88792047 | A | G | SNV | Exonic | p.F1338F | Hom | Hom | Hom | Hom | 4782430 | 87,26 | 85,098 |  |
|  |  | 88792097 | A | G | SNV | Intronic |  | Hom | Hom | Hom | Hom | 4782429 | 87,22 | 84,915 |  |
|  |  | 88793068 |  | CAGCGGGGC | Insertion | Intronic |  | Hom | Hom | Hom | Hom | 143054492 | 18,51 | 25,673 |  |
|  |  | 88793090 | A | G | SNV | Intronic |  | Hom | Hom | Hom | Hom | 4516218 | 88,558 | 81,735 |  |
|  |  | 88793103 | C | G | SNV | Intronic |  | Hom | Hom | Hom | Hom | 4424896 | 88,299 | 81,597 |  |
|  |  | 88803195 | T | C | SNV | Intronic |  | Hom | Hom | Hom | Hom | 4782410 | 99,92 | 99,846 |  |
| 19 | *KCNN4* | 44278779 | T | G | SNV | Intronic |  | Hom | Het |  | Het | 649540 | 29,413 | 23,316 |  |
|  |  | 44280837 | G | A | SNV | Intronic |  | Het | Hom | Hom | Het | 514328 | 80,751 | 85,837 |  |
| 6 | *RHAG* | 49580034 | T |  | Deletion | Intronic |  | Het | Het | Het | Het | 145988109 | 7,788 |  |  |
|  |  | 49580194 | C | T | SNV | Exonic | p.A287A | Het | Het | Het | Het | 10485291 | 7,029 | 4,351 |  |
|  |  | 49580247 | C | T | SNV | Exonic | p.V270I | Het | Het | Het | Het | 16879498* | 6,969 | 4,345 | CM991100 (DM?) |

| **Chr** | **Gene Symbol** | **Position** | **Reference Allele** | **Sample Allele** | **Variation Type** | **Gene Region** | **Protein Variant** | **Genotype** | | | | **dbSNP ID** | **1000 Genomes** | **ExAC** | **HGMD** |
| --- | --- | --- | --- | --- | --- | --- | --- | --- | --- | --- | --- | --- | --- | --- | --- |
|  |  |  |  |  |  |  |  | **RK** | **MK** | **EK** | **AK** |  |  |  |  |
| **RBC**  **enzymopathies** | | | | | | | | | | | | | | | |
| 1 | *ENO1* | 8928125 | G | T | SNV | Intronic |  | Het | Het | Het | Het | 2071412 | 34,345 | 31,778 |  |
| 9 | *AK1* | 130630233 | G | T | SNV | 3'UTR |  | Hom | Het | Het | Het | 4226 | 68,171 |  |  |
|  |  | 130630639 | A | G | SNV | Exonic | p.P175P; p.P159P | Hom | Hom | Hom | Hom | 913986 | 99,82 | 99,945 |  |
| 19 | *GPI* | 34859457 | G | A | SNV | Intronic |  | Het | Het | Het | Het | 2099099 | 68,59 | 51,239 |  |
| 7 | *NT5C3A* | 33057160 | AAC |  | Deletion | Exonic | p.F149del; p.F200del; p.F161del | Het | Het |  |  | 1227859962* |  |  |  |
|  |  | 33059407 | A | G | SNV | Intronic |  | Het | Het | Hom | Het | 2392209 | 76,897 |  |  |
| 6 | *GCLC* | 53363488 | GGCT |  | Deletion | 3'UTR |  | Het | Het | Het | Het | 138528239 | 57,728 |  |  |
|  |  | 53365019 | G | C | SNV | Intronic |  | Hom | Hom | Hom | Hom | 661346 | 99,96 | 99,809 |  |
|  |  | 53365374 | C | T | SNV | Intronic |  | Het | Het | Het | Het | 2066511 | 18,031 |  |  |
| 8 | *GSR* | 30539623 | T | G | SNV | Intronic |  | Het | Het | Het | Hom | 2250192 | 76,278 | 77,574 |  |
|  |  | 30554006 | G | A | SNV | Intronic |  | Het | Het | Het | Hom | 8190996 | 42,392 | 44,941 |  |
|  |  | 30567424 | A |  | Deletion | Intronic |  | Het | Het |  | Het | 761864201 |  | 34,427 |  |
| 20 | *GSS* | 33533736 | A | C | SNV | Intronic |  | Hom | Het | Het | Hom | 734111 | 66,434 | 61,956 |  |
| 10 | *HK1* | 71060610 | A | G | SNV | Exonic; Intronic | p.H7R | Hom | Hom | Hom | Hom | 906220* | 90,635 | 91,02 |  |
|  |  | 71060634 | G | A | SNV | Intronic |  | Hom | Hom | Hom | Hom | 906221 | 90,655 | 90,938 |  |
|  |  | 71098295 | T | A | SNV | Intronic |  | Hom | Het | Hom | Het | 7912524 | 50,18 | 53,986 |  |
|  |  | 71128875 | T | C | SNV | Intronic |  | Hom | Het | Hom | Het | 2305198 | 44,609 |  |  |
|  |  | 71142420 | G | A | SNV | Exonic | p.K469K; p.K453K; p.K485K; p.K481K; p.K516K; p.K449K; p.K480K | Hom | Het | Het | Hom | 748235 | 77,875 | 78,316 |  |
|  |  | 71144702 | G | A | SNV | Intronic |  | Hom | Het | Het | Hom | 749105 | 65,555 | 67,024 |  |
|  |  | 71152091 | T | C | SNV | Intronic |  | Het | Het | Het | Hom | 2278745 | 60,244 | 55,342 |  |
| **RBC**  **hemoglobinopathies** | | | | | | | | | | | | | | | |
| 11 | *HBB* | 5247141 | G | A | SNV | Intronic |  | Het | Het | Hom | Het | 1609812 | 71,386 |  |  |
|  |  | 5247726 | G | A | SNV | Intronic |  | Het | Het |  | Het | 7946748 | 9,924 |  |  |
|  |  | 5247733 | A | C | SNV | Intronic |  | Het | Het | Hom | Het | 7480526 | 36,901 |  |  |
|  |  | 5247791 | C | G | SNV | Intronic |  | Het | Het | Hom | Het | 10768683 | 72,005 | 76,99 |  |

**Table S1.9** Comparison between quantity of mutated genes (mutations/polymorphisms present in both brothers) obtained from Exome-Seq analysis, and respective genes deposited in the UniGene NCBI databases: CD71++++ library (Lib.8975) and GPA++ library (Lib.11923).

| **Library Name** | **Gene number** | | **Gene name** |
| --- | --- | --- | --- |
| **CD71^++++^ and GPA^++^** | 201 | *COX7A2L ATP5F1 SOD2 NOSIP RCL1 RC3H1 FARSB ASCC3 KPNA2 SCOC ANLN CHMP5 CNOT4 SRI MAP3K13 CD36 ARF4 CSDE1 NPM1 XRN2 PSMD11 USP16 CCT8 RPS4X EIF3M RPS3 LDHB NPAT ZMYND8 BTF3 SARS HPS4 RHOA QRICH1 FUBP1 RPL4 FAM188A OPA1 YIPF6 EIF4E2 MRPS7 MTHFD1 TPT1 VBP1 PPHLN1 SMARCA5 LXN RBM26 MPC2 NAA50 RHAG EPRS DDX52 THOC1 UBA3 SDHD UBE2D3 PLRG1 ATXN3 GYPA NACA GIGYF2 DNA2 MRPS9 TMEM106C SCFD1 CENPN CINP NXF1 EEF1B2 ARHGAP19 TFRC MLLT3 MRPS18C UROS PSMC1 CEP95 CLTC RAN EEF1A1 HSP90AA1 PARP1 SUCLG1 PSMA2 VPS29 UBR1 COX16 MRPL18 NIPSNAP3A EPB41 SMC2 SELK UROD SLU7 SRP14 EXOSC1 ARHGAP18 SRSF7 PRIM1 SLC31A1 HAUS1 EAPP GHITM ATP5O SP110 WDR33 RPL21 PSMB7 TACC1 NUPL2 EIF3A ATRAID RPL5 MRPL13 CMC2 EEF1D MCM7 PRC1 CDH1 UFSP2 MPHOSPH6 RPL34 NEMF ACAT2 RPAIN GLUL ANKRD36 COMMD10 GYPB CPEB4 RPL3 PCM1 TAL1 CRBN PSMA7 TMBIM6 TTF1 PSMB4 CA2 TIMMDC1 HSP90AB1 ANK1 MRPS27 GTF2B MED21 SHFM1 ATP6V1G1 DCAF13 EIF3L HBB RRM1 TMX1 SOX6 LUC7L3 RAD51AP1 ACTB MKI67 MDN1 BCCIP HBS1L RPS7 RNF10 TRAPPC4 BNIP3L AGPAT5 NDUFA9 SERINC1 CCT5 SARNP XRCC5 YBX3 ATG12 MALAT1 RAD21 CD164 CEP152 ATP5C1 MMADHC COX4I1 RPS25 AKR1C3 PSMB1 KIF22 MORF4L1 SLC25A38 SBNO1 PSME2 PDHB CAST VTA1 TBC1D23 SCAF11 CDKN3 ATP6V0E1 RPL14 UBR4 USP8 AHSP CENPF BRWD1 OAT* | |
| **GPA^++^** | 500 | *C5orf42 CD44 TRAF3IP2-AS1 DLG1 MT1B SLFN14 RAE1 TVP23C TKT ELP4 NBN QARS CLK4 HOMEZ STYK1 GTF2A1 AGFG1 RAP1A EP300 SETD2 NOS2 UGGT1 BTN2A1 SMARCC2 NDUFS1 FAM234B MIER1 SYNE2 XK RNF14 SEMA4D GABARAP AP3S2 TRIM23 SDHB LRRN1 PIKFYVE MED13L DDOST ECH1 ATG2B SAAL1 RGS16 KIAA1033 URB1 PUM3 SLC25A26 STAM FCGR2B ACTR10 STX2 FUBP3 BBS12 SQSTM1 EED ARHGEF12 MAN2A2 NAPG BIRC5 WHSC1 RAPGEF6 FBN2 DDX47 BORCS7 RPL7L1 IKZF1 UBE2I KNTC1 AP3M2 CASP10 DNAJA4 MUC16 PSMG4 RNASEK RBM23 FAS CETN3 CDK1 N4BP2L2 BTRC ACBD6 PLIN2 USP7 GMCL1 FNIP1 YME1L1 SLC26A3 COPG1 IFT140 FAM102B MZT1 NUP153 DCAF12 HSD17B11 HSD17B4 RSRC1 FBXO24 DNAH11 GSTM4 GPCPD1 CNOT1 PDCL2 SNX6 STIM1 EXOC2 KNSTRN HSF2 SRP72 CRIPT CDK17 HAUS6 INO80D ABI2 TBC1D22B QKI EZR INTS8 OFD1 DYNC1H1 ACTR1A WDR6 ASNSD1 ARMC8 WDR62 SLC22A16 NBR1 CIITA ZNF79 EMC3 HSPA8 DSC2 NGDN IMMT MARK3 ZBTB20 PPIG SUN1 BRD8 ASAH1 HEATR5B RSU1 MAD2L1 TTC21B ATP1A1 PSMF1 NRF1 TMPO TARS UIMC1 DISC1 RNF5P1 RPS2 PITPNA ILK STK38L C4orf19 SEC63 AP2M1 FTH1 PIEZO2 NPRL3 DTNB ALDH6A1 CTSB MORC3 CHRNB1 UMPS RAF1 PAQR3 TCP1 PLS1 PREX2 RTFDC1 PSMC4 EIF3D BZW1 TANK FBXL20 MAP1LC3B BEST1 DCAF11 METTL5 CORO1C ISCU MPP1 ROBO1 MYLK4 DNAJA1 CWC27 THOC7 FDFT1 ITPR1 TALDO1 ARG1 OLR1 RAD51C NELFCD ATP6V1A DGKB CDC42 ORMDL1 NINJ2 RIC8A ABI3BP MTIF3 NOL11 PCID2 GCNT2 CCNDBP1 KCNK1 NUB1 DCAF7 PARP2 RNFT2 TYMS ZSWIM6 KIAA2026 KAT8 DMXL1 PPIB ESD COQ3 PNRC1 MAT2B RBL2 ZNF677 NHEJ1 PFKM SGMS1 GART FKBP1B FAM96A ZXDC ERCC3 DNAJB6 ZNF530 ATP6V1E1 NATD1 ART4 JAZF1 GTSE1 DRG1 SRP19 PLOD2 DCAF16 PBK THEMIS2 FAM91A1 BROX ALAS1 ATG3 SKA1 FBXO7 CCND3 PDE6G GCLC HMGXB4 ERGIC3 CDC7 SEC31A GALNT10 PSMD14 GREB1L C1orf109 PSTPIP2 HMBS TRPV1 GPBP1 CASP3 ITGB3BP ALS2 HSD17B13 CUL1 RNF213 CCT7 NCKAP1 KCTD20 ZDHHC17 SLC37A4 INPP5K ZNF45 TAX1BP1 COPB2 C10orf88 BRD9 PSMD9 NDUFV3 ST3GAL2 TUBA4A WDR61 CHCHD3 MRPS10 NIP7 RB1CC1 SHCBP1 ATP5A1 SLC35F5 CNTLN MYL12B IFIT1B GLRX3 CR1L EIF1B TVP23B HERPUD1 SH3BP5L CTNNA1 SP100 TBCE LAMB1 TSC1 OARD1 BRIP1 TRIM21 UGGT2 DCAF6 DHX8 SPTY2D1 PTPRF CLECL1 SESN1 APPL2 PIP4K2A DENND1B EIF4A1 MXI1 DPY19L2P1 NELL1 TNPO3 HTT DCUN1D1 KDM4C NUP160 USF3 TBPL1 APOH TRAK2 PSAT1 C9orf84 FIGNL1 LPIN2 METTL2A SEPT2 PRG2 CCNG1 TIMM23 EPG5 ABCB10 TROVE2 RRM2 TOP2A NOX5 ACVR1 IPO7 HCFC2 JARID2 RBM22 UBE2H SPTA1 PMAIP1 PCMTD1 KRR1 KIAA1191 GUSB COPS3 FAH RABEP1 LOC283440 LRMP IFT88 LARP1B IST1 KIF15 AP3S1 GSTM3 GGCT PAN3 RIT1 ZEB2 POLR2H CLCN3 XRN1 SSR1 TMED10 ZNF507 APOBEC3C SAP30BP RRP12 RAD17 PPAT CA3 KIF24 NMI KIF13B SUGP2 ENO1 ZNF143 ADAMTS13 SLC30A5 FECH ZC3H11A C18orf25 ADAMTS3 ARG2 TGIF1 ZFAND5 REL PPP6R3 RSAD2 INSR LLPH WDR41 TATDN3 CTNNAL1 TAB3 RBBP5 ILKAP CAT SCAND2P PDS5A CUL2 PSMD13 ITCH HNRNPA3 ACSL6 HIST1H4H DAP PDGFRA CIAPIN1 HELZ EIF4A2 TMEM50A RABGAP1L TMX2 ZNHIT3 TJP2 HADHA PEX19 CFDP1 TCP11L2 NFXL1 PLB1 FNDC3B GFRA1 SPATA13 MARS NEK9 CLIP1 MAK SEMA5A C5orf22 PIP5K1B F11R TOP2B TSTD2 ORC4 PSMA6 CYB5A POU2F1 DNAJC24 BCAT1 ANKMY2 WNK1 PLEKHA5 TSFM LRCH3 GLB1 SLC14A1 STAB2 NPL STRN TMEM56 DLEU1 NARS RFC5 C21orf58 IL1R1 TDP2 POC1B CYP24A1 TMED4 SF3B3 C11orf70 ITGB5 NUDT21 ATP8B1 SUSD1 CLIC6 TP53BP2 KAT2B PRKAG1 PDLIM5 BTAF1 MDM4 UBC PNP NSUN4 SELO RNASEH1 SLC39A6* | |
| **CD71^++++^** | 453 | *SLMAP TFIP11 PIGX CCDC25 GNL3 UTP20 ORC3 RPL10A U2SURP RCBTB1 CCDC59 PAK1 ANXA1 TPX2 TECTA CLGN TRIM33 HLTF TOPBP1 CTNNBL1 PNPT1 COX15 SPATA5L1 NDUFAB1 NSMCE2 KDELC2 TRIM22 STRADB EPT1 DARS ILF2 DAD1 BMP3 AIDA SLK HNMT PNPLA4 GRPEL1 BAZ1B SDAD1 PPP1R15B LARP7 SMC3 RPS23 NUDT1 DCP1A PRDM2 ANKRD10 DAP3 PHF3 MFF STAU2 RBPJ SFR1 UFM1 FBXO11 SRSF6 TMEM222 GRB2 MGST3 CCAR1 EP400 RPL12 GCC2 MIER3 CHEK1 ENOSF1 RPLP1 PSMB6 CAMTA1 ASUN SON TRAM1 NDUFS4 PIK3CB FUS POLR2I APOL4 LARS TASP1 DDX18 PPP2R5A MED8 FAM216A LTBP1 COIL COX7A2 PSMC3IP KDM6A UFD1L TMEM14C ATP7A EMG1 PPP2R1B GLG1 EVI5* ***NT5C*** *SLC1A5 HP1BP3 RPS13 MYL6 ATP2C1 FRYL SF3A3 PARP14 NAA16 CAMSAP1 RBM6 ARIH2 MAGOHB HDGFRP3 POLD2 MCM3 LSM3 KIN CCT4 CHAF1A CENPJ TMTC3 C12orf65 QPCTL KIF20B CNDP2 SENP6 ITGB1 SRBD1 ZMYM5 WARS CCDC158 GPATCH4 ABCE1 REV3L ZNF518B TTC5 KYNU RAD54B ZCCHC7 LRR1 EPS15 BDP1 NOP16 NDUFA4 ANKRD11 WBP11 VPS11 MCM10 ANKRD17 HNRNPK SLIRP HK1 TPM1 MPHOSPH8 PRKDC PSIP1 ATM PRRC2C IK TAF1D WDR60 CEBPZ MCM5 STRAP RABGGTB PPIA CSE1L GEMIN6 FAM118A MDH2 MRPL17 SLC7A11 DERL2 SEC22B USMG5 TADA2A CCDC14 WDR26 TXNDC12 PFDN5 PPT1 SUPT16H CCNT2 POP4 CCNA2 DYNC1I2 RPS24 TADA1 ADRM1 RPL27 POLQ DYNLRB1 SMG1 VPS13D ATL3 GPD1L CCDC28A RPL36AL USP37 C8orf59 SNW1 RPP30 FBXO43 EBP MRPL22 FXR1 LSM7 TIMM8A SRRM1 NAP1L1 NDUFB3 TRAPPC13 SEPT7 EIF4B ITGA4 ARHGDIB PRPF40A CCDC167 CPNE8 PSMA4 ZC3H13 CCDC174 DIAPH3 CTR9 HNRNPH1 GRB10 LANCL3 GLO1 TRPM7 NUCKS1 PPIL3 EXOC4 OSTC EIF5B RPL36 SACS LRP6* ***NT5C3A*** *METTL14 MRPL32 ACADM CMSS1 CAPZA1 OPTN TOMM6 AHSA1 TFB2M HMGN2 NME2 CPSF1 KIDINS220 FNIP2 PYGL G3BP2 RPS10 GADD45A FAM136A GTF2A2 HIBCH CYP20A1 TDRD3 BIVM BLVRA SNAP23 CDK13 SNX9 ANKRD36C GLMN RAB3D SRPK2 AGAP6 DHX57 GLT8D1 RIOK2 CALU ITPA TDG SAP18 TRDMT1 SKIV2L2 FCF1 SMARCA4 FIP1L1 CKAP2 BOD1L1 RPS12 PABPC4 TMEM245 DIAPH1 TCERG1 AKAP9 RTCA HDAC8 SF3B1 RNASEH2B MTAP RFC1 TSG101 TUBA1C RBBP7 FAM69A TXNRD1 SNRNP25 TNNI3K MTFP1 FAM172A CCT3 RAD50 SMU1 HSP90B1 NCL PRPF18 GUF1 WDHD1 TIMM8B TMCO1 TGS1 RPL27A YTHDF2 CEP192 RPL9 RB1 GOLIM4 WRN METAP2 PET100 IRF2 JMJD1C DDX10 PPP2CA BAZ1A INPP5D ARID4A ESCO2 NVL TM9SF3 TAF8 DTL NUDT13 SRFBP1 CREBRF ARL6IP4 TM7SF3 PSMC5 LRRFIP2 CDC123 INTS4 TTN ACP1 PPA2 CIT IER3IP1 PPIP5K2 DLD TRAF3IP3 TCEB2 PHF20L1 HMMR PTBP2 MRPS21 MGAT3 RPL37A QSER1 ADIPOR2 EBNA1BP2 ANKIB1 SART3 METTL13 HOOK1 SNRPD3 LAP3 TSR1 RPL11 ELMSAN1 GMNN RSRC2 SPCS2 TBRG4 FAM104B NNT RNMT MRPL42 MICALL2 CENPK KIAA0430 POP5 NDUFB2 PLEKHG1 UBR2 MINOS1 PPIE NUDT5 SEPT11 UBE2V1 EIF3E ADSS BCL11A RPL31 PSMB3 RPN2 MTIF2 PPP2R5E GMPS SFXN4 HIST1H1D SNRNP40 WDYHV1 LPIN1 CCP110 LIN7C APC RSL1D1 RBM25 TMSB4X MECR TPR TTC4 MAMDC2 REXO2 CNOT11 GTF2H3 RANBP2 LOC100288152 NAT1 ATIC TOMM7 PNN MRPL30 TXNDC17 MARVELD2 SCAF4 CSNK2A1 NCAPG RBM28 ATPAF2 SUCLA2 IDH3B DAAM1 KTN1 SMARCE1 DNAH5 TMEM256 RCN2 GINS2 LYPLAL1 ATP5G1 PPWD1 EAF2 GNL2 ACTR2 LBR RAP1GDS1 TOP1 CDYL CDC25A* | |

**Table S1.10** List of variants whose frequency does not exceed 10% identified in the studied family using the WES analysis. The list is restricted to a recessive pattern of inheritance and/or potential compound heterozygotes extended with a mutation detected in the genes (het – heterozygotic; hom – homozygotic; no-call - all alleles are partially or fully no-called; het-alt - diploid, heterozygous, fully called locus where both alleles differ from the reference). * The list includes genes correlated with the anemia phenotype (according to *GeneAnalytics^TM^*) in which low frequency changes were detected in both patients regardless of the inheritance pattern (≤10%). ** In one case the inheritance pattern for selected family members turned out to be different from the results obtained by WES analysis. Polymorphism rs750339397 (*SEC23B*) was absent in WES analysis for one sample (EK) but was detected in heterozygous genotypes using the Sanger method. These data were corrected in the table.

| **Chr** | **Position** | **Gene Symbol** | **Transcript Variant** | **Protein Variant** | **Genotype** | | | |  | **Translation Impact** | **dbSNP ID** |
| --- | --- | --- | --- | --- | --- | --- | --- | --- | --- | --- | --- |
|  |  |  |  |  | **RK** | **MK** | **EK** | **AK** |  |  |  |
| 1 | 21011355 | *KIF17* | c.2178A>G; c.1878A>G | p.A626A;p.A726A | Het | Het | Het |  |  | synonymous | 61750850 |
| 1 | 21012609 |  | c.1949C>T; c.1649C>T | p.P650L; p.P550L | Het | Het |  |  |  | missense | 41310420 |
| 1 | 114940633 | *TRIM33* | c.3121-151delT; c.3121-21delT |  | Het | Het | Het |  |  |  | 5777192 |
| 1 | 114963113 |  | c.2061+945T>C |  | Het | Het |  |  |  |  | 7512590 |
| 1 | 115053498 |  | c.200T>C | p.V67A | Het | Het | Het |  |  | missense | 6691166 |
| 1 | 120469077 | *NOTCH2* | c.4005+45A>G |  | Hom | Hom | Het | Het |  |  | 17258579 |
| 1 | 120612040 |  | c.-21_-20insCGGCGGAGG |  | Het | Het | Het | Het |  |  | 782658834 |
| 1 | 144922224 | *PDE4DIP* | c.944G>A; c.6089+97468C>T; c.1142G>A; c.1433G>A | p.R315Q; p.R478Q; p.R381Q | Het | Het |  |  |  | missense | 782617784 |
| 1 | 144994902 |  | c.-171C>T; c.442-201C>T; c.39+54C>T; c.-66-201C>T; c.6089+170146G>A; c.229-201C>T |  | Het | Het |  |  |  |  | 4649499 |
| 1 | 145014187 |  | c.441+1673delT; c.6089+189431delA; c.228+1673delT |  | Het | Het | Het | Het |  |  | 67011214 |
| 1 | 145014210 |  | c.228+1650C>T; c.441+1650C>T; c.6089+189454G>A |  | Het | Het | Het | Het |  |  | 1778710 |
| 2 | 175614909 | *CHRNA1* | c.779-14_779-12delTTT; c.854-14_854-12delTTT |  | Het-Alt | Het-Alt |  |  |  |  | 773734065 |
| 2 | 175614910 |  | c.854-14_854-13delTT; c.779-14_779-13delTT |  | Het-Alt | Het-Alt | Het | Het |  |  | 771673267 |
| 2 | 238234338 | *COL6A3* | c.8740A>C; c.7537A>C; c.9358A>C | p.T2914P; p.T3120P; p.T2513P | Het | Het |  |  |  | missense | 141050617 |
| 2 | 238243285 |  | c.7392C>T; c.8595C>T; c.9213C>T | p.H2464H; p.H2865H; p.H3071H | Het | Hom | Het | Het |  | synonymous | 2270671 |
| 2 | 238253149 |  | c.6894C>T; c.7512C>T; c.5691C>T | p.N1897N; p.N2504N; p.N2298N | Het | Hom | Het | Het |  | synonymous | 2646258 |
| 4 | 6607046 | *MAN2B2* | c.1651A>G; c.1804A>G | p.I551V; p.I602V | Het | Het |  | Het |  | missense | 144650979* |
| 4 | 6610889 |  | c.1870G>A; c.1717G>A | p.G573R; p.G624R | Het | Het | Het |  |  | missense | 61733402* |
| 4 | 7684544 | *SORCS2* | c.1416C>T | p.N472N | Het | Het |  | Het |  | synonymous | 35306661 |
| 4 | 7691375 |  | c.1591+60A>G |  | Het | Het |  | Het |  |  | 76278311 |
| 4 | 7719691 |  | c.2253-48C>T |  | Het | Het | Het |  |  |  | 201538318 |
| 5 | 79029762 | *CMYA5* | c.5174C>T | p.S1725L | Het | Het | Het | Het |  | missense | 17254174 |
| 5 | 79032283 |  | c.7695C>T | p.A2565A | Het | Het | Het | Het |  | synonymous | 141532983 |
| 5 | 79035139 |  | c.10551A>G | p.K3517K | Het | Het |  |  |  | synonymous | 1489606668 |
| 5 | 150502612 | *ANXA6* | c.1043-40C>T; c.1139-40C>T |  | Het | Het |  |  |  |  | 41290567 |
| 5 | 150519660 |  | c.13+54G>A; c.109+54G>A |  | Het | Het | Het | Het |  |  | 72790141 |
| 5 | 150527532 |  | c.-25-45G>T |  | Het | Het | Het | Het |  |  | 41290573 |
| 5 | 150663655 | *SLC36A3* | c.1047G>A; c.924G>A | p.K308K; p.K349K | Het | Het | Het | Het |  | synonymous | 17660011 |
| 5 | 150664343 |  | c.709-71C>G; c.832-71C>G |  | Het | Het |  |  |  |  | 10476766 |
| 5 | 150666946 |  | c.692G>A; c.569G>A | p.R231H; p.R190H | Het | Het | Het | Het |  | missense | 17660042 |
| 5 | 150666962 |  | c.676C>T; c.553C>T | p.P226S; p.P185S | Het | Het |  |  |  | missense | 12520516 |
| 5 | 150908812 | *FAT2* | c.9953G>A | p.R3318Q | Het | Het | Het | Het |  | missense | 7718054 |
| 5 | 150946780 |  | c.1713G>T | p.G571G | Het | Het | Het | Het |  | synonymous | 35225143 |
| 5 | 150948016 |  | c.477G>A | p.E159E | Het | Het |  |  |  | synonymous | 3734062 |
| 6 | 25435655 | *LRRC16A* | c.250-56delT |  | Hom | Hom | Het | Het |  |  | 34314506 |
| 6 | 25472766 |  | c.874+17_874+19delATT |  | Het | Het |  | Het |  |  | 570657994 |
| 6 | 25510697 |  | c.1478-38_1478-37delAT |  | Het | Het |  | Het |  |  | 139587245 |
| 6 | 49580034 | *RHAG** | c.945+76delA |  | Het | Het | Het | Het |  |  | 145988109 |
| 6 | 49580194 |  | c.861G>A | p.A287A | Het | Het | Het | Het |  | synonymous | 10485291 |
| 6 | 49580247 |  | c.808G>A | p.V270I | Het | Het | Het | Het |  | missense | 16879498 |
| 6 | 51524403 | *PKHD1* | c.10521C>T | p.H3507H | Het | Het |  |  |  | synonymous | 34460237 |
| 6 | 51930886 |  | c.779-12_779-11insT |  | Hom | Hom | Het | Het |  |  | 5876252 |
| 6 | 52993002 | *GCM1* | c.*2C>T |  | Het | Het |  | Het |  |  | 13200319 |
| 6 | 53010463 |  | c.-33A>G |  | Het | Het |  |  |  |  | 45476600 |
| 6 | 56485023 | *DST* | c.3809A>G; c.3318+4305A>G; c.4416+4305A>G; c.4830+4305A>G; c.4296+4305A>G | p.K1270R | Het | Het | Het | Het |  | missense | 35497571 |
| 6 | 56496649 |  | c.3450+32T>G; c.2352+32T>G; c.3864+32T>G; c.3330+32T>G |  | Het | Het |  |  |  |  | 10456737 |
| 6 | 65300516 | *EYS* | c.5244A>C | p.L1748F | Het | Het | Het | Het |  | missense | 57312007 |
| 6 | 66005857 |  | c.1922A>T | p.E641V | Het | Het |  |  |  | missense | 17411795 |
| 6 | 90438631 | *MDN1* | c.5349+19C>A |  | Het | Het |  |  |  |  | 76752399 |
| 6 | 90450101 |  | c.4449-4T>G |  | Het | Het | Het | Het |  |  | 12203928 |
| 6 | 90466093 |  | c.2719G>A | p.V907I | Het | Het |  |  |  | missense | 1315382275 |
| 6 | 129571335 | *LAMA2* | c.1861C>T | p.L621F | Het | Het |  |  |  | missense | 139093923 |
| 6 | 129663464 |  | c.4312-24_4312-22delCTT |  | Het | Het | Het | Het |  |  | 200038968 |
| 6 | 131902329 | *MED23; ARG1* | c.306-30G>A; c.330-30G>A; c.4095+6520C>T; c.4077+6520C>T |  | Het | Het |  |  |  |  | 41285336 |
| 6 | 131948470 | *MED23* | c.159+66delT |  | Het | Het | Het | Het |  |  | 60163644 |
| 6 | 131948816 |  | c.40-5_40-4insT |  | Het | Het |  |  |  |  | 754667101 |
| 6 | 152443761 | *SYNE1* | c.26060G>A; c.26204G>A | p.R8735Q; p.R8687Q | Het | Het | Het |  |  | missense | 2295192 |
| 6 | 152489180 |  | c.23628-3720C>T; c.23415-3720C>T |  | Het | Het | Het |  |  |  | 78594564 |
| 6 | 152577752 |  | c.18891+17A>G; c.19104+17A>G |  | Het | Het |  | Het |  |  | 17082389 |
| 6 | 152603139 |  | c.17995-24A>G; c.18208-24A>G |  | Het | Het |  | Het |  |  | 9478310 |
| 6 | 152746593 |  | c.5211T>A; c.5190T>A | p.D1730E; p.D1737E | Het | Het | Het |  |  | missense | 111250109 |
| 6 | 152763258 |  | c.3960A>G; c.3981A>G | p.T1327T; p.T1320T | Het | Het | Het |  |  | synonymous | 138705766 |
| 6 | 152770645 |  | c.3525+23C>T; c.3504+23C>T |  | Het | Het | Het |  |  |  | 17366321 |
| 6 | 152771849 |  | c.3327C>T; c.3306C>T | p.H1102H; p.H1109H | Het | Het | Het |  |  | synonymous | 17082701 |
| 6 | 152777095 |  | c.2653T>G; c.2674T>G | p.L885V; p.L892V | Het | Het | Het |  |  | missense | 17082709 |
| 6 | 152793628 |  | c.1351-80G>A; c.1372-80G>A |  | Het | Het | Het |  |  |  | 4530871 |
| 6 | 159653330 | *FNDC1* | c.1786G>A | p.G596S | Hom | Het |  | Het |  | missense | 117860656 |
| 6 | 159653544 |  | c.2000G>A | p.R667Q | Hom | Hom | Het | Het |  | missense | 139265083 |
| 7 | 5369522 | *TNRC18* | c.6147+2731C>G |  | Hom | Het | Het | Het |  |  | 73051980 |
| 7 | 5416426 |  | c.2608+52G>A |  | Het | Het |  |  |  |  | 73055894 |
| 7 | 5434107 |  | c.307C>T | p.P103S | Het | Het |  |  |  | missense | 73057725 |
| **7** | **33057160** | ***NT5C3A**** | **c.444_446delGTT; c.480_482delGTT; c.597_599delGTT** | **p.F149del; p.F200del; p.F161del** | **Het** | **Het** |  |  |  | **in-frame** | **1227859962** |
| 7 | 38765883 | *VPS41* | c.2453G>A; c.2528G>A | p.R843H; p.R818H | Het | Het |  | Het |  | missense | 1059508 |
| 7 | 38810880 |  | c.1129-27_1129-26insT; c.1054-27_1054-26insT |  | Het | Het | Het |  |  |  | 748511011 |
| 8 | 95143138 | *CDH17* | c.2250T>C | p.G750G | Het | Het |  | Het |  | synonymous | 2513797 |
| 8 | 95186511 |  | c.425-23delT |  | Het | Het | Het |  |  |  | 775842426 |
| 8 | 95189780 |  | c.285+35A>G |  | Het | Het |  | Het |  |  | 79317393 |
| 8 | 99033441 | *MATN2* | c.1828G>A | p.V610I | Het | Het | Het |  |  | missense | 61729731 |
| 8 | 99046376 |  | c.2758+489_2758+490insT; c.2815+489_2815+490insT |  | Het | Het |  | Het |  |  | 35312215 |
| 8 | 99046383 |  | c.2758+496_2758+497insT; c.2815+496_2815+497insT |  | Het | Het |  | Het |  |  | 368572002 |
| 8 | 120431506 | *NOV* | c.698G>A | p.R233H | Hom | Hom | Het | Het |  | missense | 11538929 |
| 9 | 19086619 | *HAUS6* | c.699+112_699+113delTT |  | Het | Het |  | No-Call |  |  | unknown |
| 9 | 19087196 |  | c.585-42C>T |  | Het | Het | Het |  |  |  | 7860085 |
| 9 | 35708425 | *TLN1* | c.4383A>G | p.L1461L | Het | Het |  | Het |  | synonymous | 35461988 |
| 9 | 35717135 |  | c.2458+8A>G |  | Het | Het | Het |  |  |  | 142090240 |
| 10 | 104118983 | *GBF1* | c.1012-41G>C; c.1012-44G>C |  | Het | Het |  | Het |  |  | 41293036 |
| 10 | 104120732 |  | c.1390-47delA; c.1393-47delA |  | Het | Het | Het |  |  |  | 375688888 |
| 10 | 104140350 |  | c.5065G>A; c.5077G>A; c.5068G>A | p.G1690S; p.G1693S; p.G1689S | Het | Het |  | Het |  | missense | 11191274 |
| 11 | 396915 | *PKP3* | c.414C>G; c.459C>G | p.N138K; p.N153K | Het-Alt | Het-Alt | Het |  |  | missense | 12419281 |
| 11 | 397331 |  | c.830G>C; c.875G>C | p.R277P; p.R292P | Het | Het |  | Het |  | missense | 200371913 |
| 11 | 1021268 | *MUC6* | c.3536A>G | p.N1179S | Het | Het | Het |  |  | missense | 113451874 |
| 11 | 1023496 |  | c.3526+13C>G |  | Het | Het |  | Het |  |  | 12793770 |
| 11 | 1026084 |  | c.2604C>T | p.C868C | Het | Het |  | Het |  | synonymous | 12801568 |
| 11 | 1029135 |  | c.1291G>A | p.E431K | Het | Het | Het |  |  | missense | 59763899 |
| 11 | 3111782 | *OSBPL5* | c.2194+6C>T; c.2398+6C>T |  | Het | Het | Het |  |  |  | 2412134 |
| 11 | 3150391 |  | c.-13G>A |  | Het | Het |  | Het |  |  | 2277300 |
| 11 | 6411936 | *SMPD1* | c.138_143delGCTGGC | p.L47_A48del | Hom | Hom | Het | Het |  | in-frame | 3838786 |
| 11 | 8111121 | *TUB* | c.39-36C>T; c.204-36C>T |  | Het | Het | Het |  |  |  | 1317500 |
| 11 | 8122228 |  | c.1215+80A>G; c.1380+80A>G |  | Het | Het |  | Het |  |  | 12796222 |
| 12 | 80613571 | *OTOGL* | c.209-23delT |  | Het | Het |  |  |  |  | 753698992 |
| 12 | 80729949 |  | c.4573+29delT |  | Het | Het | Het | Het |  |  | 753091055 |
| 12 | 123969828 | *RILPL1* | c.974+352T>C |  | Hom | Hom | Het | Het |  |  | 11522343 |
| 12 | 129566270 | *TMEM132D* | c.1923+34T>G |  | Het | Het | Het |  |  |  | 12578725 |
| 12 | 129566285 |  | c.1923+19delA |  | Het | Het | Het | Het |  |  | 34663182 |
| 12 | 129566406 |  | c.1821G>A | p.T607T | Het | Het | Het |  |  | synonymous | 79031518 |
| 12 | 130015535 |  | c.1115+68_1115+69insT |  | Het | Hom | Het | Het |  |  | 778001774 |
| 12 | 130184612 |  | c.711G>A | p.G237G | Het | Het |  |  |  | synonymous | 140064887 |
| 12 | 133197789 | *P2RX2* | c.906-52G>A; c.804-52G>A; c.630-52G>A; c.834-52G>A; c.690-52G>A |  | Hom | Hom |  | Het |  |  | 191630133 |
| 12 | 133202816 | *POLE* | c.6418G>A | p.E2140K | Hom | Hom |  | Het |  | missense | 5745066 |
| 13 | 76210772 | *LMO7* | c.-22C>A; c.225+13316C>A |  | Het | Het | Het |  |  |  | unknown |
| 13 | 76335109 |  | c.408A>G; c.-448A>G; c.252A>G | p.G136G; p.G84G | Het | Het | Het |  |  | synonymous | 17706535 |
| 13 | 76383408 |  | c.1100+89A>T; c.1946+89A>T; c.1247+89A>T |  | Het | Het |  | Het |  |  | 151237725 |
| 13 | 77635245 | *MYCBP2* | c.13036+59G>A |  | Hom | Hom | Het | Het |  |  | 2285388 |
| 13 | 77663138 |  | c.10554G>A | p.P3518P | Hom | Hom | Het | Het |  | synonymous | 34700794 |
| 13 | 77699596 |  | c.7892A>G | p.N2631S | Hom | Hom | Het | Het |  | missense | 34474844 |
| 13 | 77732208 |  | c.6634C>T | p.L2212L | Hom | Hom | Het | Het |  | synonymous | 34982494 |
| 13 | 77740709 |  | c.6115-20A>G |  | Hom | Hom | Het | Het |  |  | 3742103 |
| 13 | 77852856 |  | c.748+37G>A |  | Hom | Hom | Het | Het |  |  | 77595208 |
| 13 | 99484109 | *DOCK9* | c.4246-69C>G; c.4249-69C>G |  | Hom | Hom | Het | Het |  |  | 17709432 |
| 13 | 99498847 |  | c.4062-602delA; c.4065-602delA |  | Het | Het | Het |  |  |  | unknown |
| 13 | 99554548 |  | c.1374G>A; c.1377G>A | p.P459P; p.P458P | Het | Het |  | Het |  | synonymous | 75205686 |
| 13 | 99556863 |  | c.1038+23A>C; c.1035+23A>C |  | Het | Het | Het |  |  |  | 17783411 |
| 13 | 109518578 | *MYO16* | c.1413T>C; c.1347T>C | p.I471I; p.I449I | Het | Het | Het |  |  | synonymous | 9559428 |
| 13 | 109609951 |  | c.1860-85G>A; c.1926-85G>A |  | Het | Het |  | Het |  |  | 9559450 |
| 16 | 5055865 | *SEC14L5* | c.1303-50_1303-49delAA |  | Het-Alt | Het-Alt |  |  |  |  | 768199486 |
| 16 | 5055866 |  | c.1303-49delA |  | Het-Alt | Het-Alt | Het-Alt | Het-Alt |  |  | 753200763 |
| 16 | 14029033 | *ERCC4** | c.1244G>A | p.R415Q | Het | Het | Het |  |  | missense | 1800067 |
| 16 | 14031818 |  | c.1904+103G>A |  | Het | Het |  | Het |  |  | 1035109661 |
| 16 | 19883364 | *GPRC5B* | c.1197C>T; c.804C>T | p.N268N; p.N399N | Het | Het | Het |  |  | synonymous | 61742688 |
| 16 | 19883550 |  | c.618C>T; c.1011C>T | p.Y206Y; p.Y337Y | Het | Het |  | Het |  | synonymous | 142444695 |
| 16 | 69201050 | *UTP4* | c.1906C>T | p.R636C | Het | Het |  | Het |  | missense | 61185783 |
| 16 | 69202908 |  | c.*68A>G |  | Het | Het | Het |  |  |  | 74886619 |
| 16 | 71956592 | *IST1* | c.315+9_315+10insAG; c.798+9_798+10insAG; c.759+9_759+10insAG |  | Het | Het | Het |  |  |  | 75418123 |
| 16 | 71958025 |  | c.853-647C>G; c.892-647C>G; c.409-647C>G; c.760-647C>G; c.853-651C>G |  | Het | Het |  | Het |  |  | 74891946 |
| 16 | 72032166 | *PKD1L3* | c.418+5G>A |  | Het | Het |  | Het |  |  | 376094120 |
| 16 | 72033580 |  | c.295+3A>T |  | Het | Het | Het |  |  |  | 60493669 |
| 17 | 21202310 | *MAP2K3* | c.165+72G>A; c.78+72G>A |  | Het | Het | Het | Het |  |  | 77343934 |
| 17 | 21202311 |  | c.165+73C>T; c.78+73C>T |  | Het | Het | Het | Het |  |  | 75476957 |
| 17 | 21202316 |  | c.165+78T>C; c.78+78T>C |  | Het | Het | Het | Het |  |  | 78463537 |
| 17 | 21202318 |  | c.165+80G>T; c.78+80G>T |  | Het | Het | Het | Het |  |  | 77591777 |
| 17 | 21207697 |  | c.569-41G>A; c.482-41G>A |  | Het | Het |  |  |  |  | 201691897 |
| 17 | 21208347 |  | c.697-16C>A; c.610-16C>A |  | Het | Het | Het | Het |  |  | 4559960 |
| 17 | 21217397 |  | c.874-62A>G; c.961-62A>G |  | Het | Het | Het | Het |  |  | 1657685 |
| 17 | 21217400 |  | c.961-59G>A; c.874-59G>A |  | Het | Het | Het | Het |  |  | 1622401 |
| 17 | 56798128 | *RAD51C** | c.859A>G | p.T287A | Het | Het |  |  |  | missense | 28363317 |
| 18 | 44561619 | *TCEB3B; KATNAL2* | c.-94-17632G>C; c.17C>G | p.T6S | Het | Hom |  | Het |  | missense | 61738602 |
| 18 | 44626630 | *KATNAL2* | c.1164T>G | p.T388T | Het | Het |  |  |  | synonymous | 2289130 |
| 18 | 44656759 | *HDHD2* | c.311-60T>A |  | Het | Het |  |  |  |  | 78904615 |
| 18 | 67718690 | *RTTN* | c.5280G>A | p.P1760P | Het | Het | Het |  |  | synonymous | 186543005 |
| 18 | 67863844 |  | c.734A>G | p.K245R | Het | Het |  | Het |  | missense | 17082206 |
| 18 | 67866635 |  | c.578+15G>A |  | Het | Het |  | Het |  |  | 17808334 |
| 18 | 67873001 |  | c.-107C>T |  | Het | Het |  | Het |  |  | 73966851 |
| 18 | 77097360 | *ATP9B* | c.2194A>T | p.M732L | Hom | Hom | Het | Het |  | missense | 585033 |
| 20 | 18505564 | *SEC23B** | c.604-15delT; c.550-15delT |  | Het | Het | Het** | Het |  |  | 750339397 |
| 21 | 46554605 | *ADARB1* | c.-48+6117insT |  | Het-Alt | Het-Alt |  |  |  |  | 11369444 |
| 21 | 46554605 |  | c.-48+6117delT |  | Het-Alt | Het-Alt | Het | Het |  |  | 200755576 |
| 22 | 44465036 | *PARVB* | c.-44-24772C>T; c.113-24772C>T; c.-6C>T; c.212-24772C>T |  | Het | Het | Het |  |  |  | 73888833 |
| 22 | 44532259 | *PARVB* | c.733-81C>T; c.478-81C>T; c.523-81C>T; c.634-81C>T |  | Het | Het |  | Het |  |  | 17584043 |
| 22 | 50297035 | *ALG12* | c.*450_*451delAG |  | Het | Het |  | Het |  |  | 16445 |
| 22 | 50297888 |  | c.1177A>G | p.I393V | Het | Het |  | Het |  | missense | 3922872 |
| 22 | 50298118 |  | c.1029G>A | p.A343A | Het | Het | Het |  |  | synonymous | 62233155 |
| 22 | 50307184 |  | c.163-19C>A |  | Het | Het |  | Het |  |  | 11705497 |
| 22 | 50962078 | *SCO2*; NCAPH2* | c.*274G>T; c.763C>A | p.R255R | Het | Het | Het |  |  | synonymous | 112793292 |
| 22 | 50964255 | *SCO2*; TYMP* | c.-398G>A; c.-14+175G>A; c.1393G>A; c.-14+420G>A; c.-368G>A; c.1408G>A | p.A465T; p.A470T | Het | Het |  | Het |  | missense | 112723255 |
| X | 2833769 | *ARSD* | c.864-36A>G |  | Het | Het |  | Het |  |  | 199688335 |
| X | 2835985 |  | c.723C>T | p.F241F | Het | Het |  | Het |  | synonymous | 748243474 |
| X | 2835993 |  | c.715C>T | p.L239L | Het | Het |  | Het |  | synonymous | 755296450 |
| X | 7066168 | *PUDP* | c.-14C>T |  | Hom | Hom | Het |  |  |  | 187333600 |
| X | 9682850 | *TBL1X* | c.1453-92C>T; c.1606-92C>T |  | Hom | Hom | Het |  |  |  | 56327515 |
| X | 10047921 | *WWC3* | c.377+77A>G |  | Hom | Hom | Het |  |  |  | 141531107 |
| X | 13785455 | *OFD1* | c.2757+52C>T |  | Hom | Hom | Het |  |  |  | 189935382 |
| X | 13799089 | *GPM6B* | c.349-981C>T; c.406-981C>T; c.526-981C>T |  | Hom | Hom | Het |  |  |  | 774667301 |
| X | 14027247 | *GEMIN8* | c.514G>A | p.V172M | Hom | Hom | Het |  |  | missense | 145874697 |
| X | 17043368 | *REPS2* | c.673+60C>T; c.670+60C>T |  | Hom | Hom | Het |  |  |  | 9887218 |
| X | 18972497 | *PHKA2* | c.112G>C | p.E38Q | Hom | Hom | Het |  |  | missense | 17313469 |
| X | 20070968 | *MAP7D2* | c.484+3830C>G; c.595+28C>G; c.463+28C>G |  | Hom | Hom | Het |  |  |  | 139258333 |
| X | 20253005 | *RPS6KA3* | c.70-73delT |  | Het | Het |  |  |  |  | 758120780 |
| X | 24844699 | *POLA1* | c.3699C>A | p.V1233V | Hom | Hom | Het |  |  | synonymous | 11573423 |
| X | 40460183 | *ATP6AP2* | c.858+50_858+51insAA |  | Hom | Hom | Het-Alt | Het-Alt |  |  | 10590549 |
| X | 91147704 | *PCDH11X* | c.3033+13432delT |  | Het | Het |  |  |  |  | 35310035 |
| X | 153218148 | *HCFC1* | c.4759C>T | p.L1587F | Hom | Hom |  |  |  | missense | 1557112939 |
| X | 153221575 |  | c.2856+67C>T |  | Hom | Hom |  |  |  |  | 56289369 |

**Table S1.11** Number of variants potentially significant for the HA phenotype identified by WES and filtered by *Ingenuity Variant Analysis* in the studied family members.

|  | **Patient (RK and MK series A/B) vs asymptomatic family members (series C)** | | **Patient (RK and MK series A/B) vs asymptomatic family members (series B)** | |
| --- | --- | --- | --- | --- |
|  | **Changes present  in studied family** | **Changes present  in both patients** | **Changes present  in studied family** | **Changes present  in both patients** |
| Common Variants | 2072 | 429 | 1818 | 420 |
| Predicted Deleterious | 564 | 98 | 506 | 98 |
| Genetic Analysis | 94 | 16 | 92 | 16 |
| Biological Context | 9 | 3 | 8 | 3 |

**Table 1.12** Selectively enriched biological pathways were identified with gene set enrichment analysis. The set of genes identified using the *GeneAnalytics^TM^* web server was associated with 20 pathways resulting in the identification of seven genes with the highest score associated with four bold phenotypes correlated with pyrimidine and purine metabolism. The list of 82 genes was restricted to a recessive pattern of inheritance and/or potential compound heterozygotes. Gene variants were identified in both patients with HA by WES analysis according to their frequency does not exceed 10 % (see TABLE S1.8). Additionally, the list includes genes correlated with the anemia phenotype in which low frequency (≤10%) changes were detected in both patients regardless of the inheritance pattern. All genes get medium scores in the Pathways matching analysis.

| **Score** | **SuperPath Name** | **SuperPath Total Genes** | **SuperPath Matched Genes** | **Matched Genes (Symbols)** |
| --- | --- | --- | --- | --- |
| 12.45 | **Pyrimidine Deoxyribonucleosides Degradation** | 5 | 2 | ***TYMP, SCO2*** |
| 10.24 | **Pyrimidine Metabolism** | 47 | 3 | ***TYMP, NT5C3A, PUDP*** |
| 9.18 | **Purine Metabolism (KEGG)** | 223 | 5 | ***ADARB1, TYMP, POLA1, POLE, NT5C3A*** |
| 8.94 | Organelle Biogenesis and Maintenance | 341 | 6 | *HAUS6, GBF1, KIF17, HCFC1, TBL1X, OFD1* |
| 8.44 | Homologous DNA Pairing and Strand Exchange | 73 | 3 | *ERCC4, RAD51C, POLE* |
| 7.72 | Focal Adhesion | 283 | 5 | *LAMA2, TLN1, COL6A3, MAP2K3, PARVB* |
| 7.46 | Mismatch Repair | 29 | 2 | *POLA1, POLE* |
| 7.27 | **Purine Metabolism (REACTOME)** | 98 | 3 | ***TYMP, NT5C3A, PUDP*** |
| 7.03 | MRNA Editing- A to I Conversion | 2 | 1 | *ADARB1* |
| 6.86 | Smooth Muscle Contraction | 36 | 2 | *ANXA6, TLN1* |
| 6.65 | Cell Cycle, Mitotic | 622 | 7 | *HAUS6, SYNE1, RAD51C, POLA1, OFD1, POLE, NCAPH2* |
| 6.51 | Chks in Checkpoint Regulation | 224 | 4 | *ERCC4, POLA1, SEC23B, POLE* |
| 6.50 | RhoA Signaling Pathway | 41 | 2 | *TLN1, MAP2K3* |
| 6.37 | Cell Cycle Control of Chromosomal Replication | 43 | 2 | *POLA1, POLE* |
| 6.05 | Cell Junction Organization | 134 | 3 | *CDH17, DST, PARVB* |
| 6.03 | Metabolism | 2544 | 17 | *ARG1, ARSD, SMPD1, TBL1X, MAN2B2, MED23, PHKA2, OSBPL5, RPL10, TYMP, RHAG, POLA1, SCO2, ALG12, POLE, NT5C3A, PUDP* |
| 5.72 | Lysosomal Oligosaccharide Catabolism | 5 | 1 | *MAN2B2* |
| 5.61 | Agrin Interactions at Neuromuscular Junction | 57 | 2 | *CHRNA1, LAMA2* |
| 5.52 | Mitochondrial Gene Expression | 59 | 2 | *HCFC1, TBL1X* |
| 5.46 | Factors Involved in Megakaryocyte Development and Platelet Production | 157 | 3 | *DOCK9, CARMIL1, RAD51C* |

**Table S1.13** List of variants identified in cDNA using Sanger sequencing (het – heterozygotic; hom – homozygotic).

| **dbSNP ID** | **Gene Symbol** | **Transcript Variant** | **Protein Variant** | **RK** | **MK** | **EK** | **AK** |
| --- | --- | --- | --- | --- | --- | --- | --- |
| 34474844 | *MYCBP2* | c.7892A>G | p.N2631S | hom | hom | het | het |
| 1557112939 | *HCFC1* | c.4759C>T | p.L1587F | hom | hom | - | - |
| 61733402 | *MAN2B2* | c.1651A>G; c.1804A>G | p.I551V; p.I602V | het | het | het | - |
| 144650979 |  | c.1870G>A; c.1717G>A | p.G573R; p.G624R | het | het | - | het |
| 2287622 | *ABCB11* | c.1331T>C | p.V444A | hom | hom | het | het |
| 5980322 | *CTPS2* | c.720+37C>T |  | hom | hom | het | - |
| 199636910 | *PELP1* | c.2105T>C; c.2696T>C | p.V702A; p.V899A | het | het | - | - |
| 200062536 |  | c.1570A>G; c.2161A>G | p.M524V; p.M721V | het | het | - | - |
| 145874697 | *GEMIN8* | c.514G>A | p.V172M | hom | hom | het | - |
| **1227859962** | ***NT5C3A*** | **c.444_446delGTT** | **p.F149del** | **hom** | **hom** | **-** | **-** |
| 2230148 | *XK* | c.*89A>T |  | hom | hom | het | - |
| 5745066 | *POLE* | c.6418G>A | p.E2140K | hom | hom | - | het |
| 11538929 | *NOV* | c.698G>A | p.R233H | het | hom | het | het |
| 812808 | *DOCK9* | c.6210-3A>G; |  | hom | hom | het | het |
| 187333600 | *PUDP* | c.-14C>T |  | hom | hom | hom | - |
| 3838786 | *SMPD1* | c.138_143delGCTGGC | p.L47_A48del | hom | hom | het | het |
| 200652126 | *CLGN* | c.1309G>T | p.D437Y | het | het | - | - |

**Table S1.14** Polymorphisms/mutations present in studied family members located in the *NT5C3A* gene identified using the Sanger method (het – heterozygotic; hom – homozygotic). *accessed on 25 November 2021

| **Product** | ***NT5C3A*  transcript  variant 4** | **Protein  isoform 3** | **Inheritance  patients/ asymptomatic  family members** | | | | **dbSNP ID** | **Frequency of change***  **MAF/Minor**  **Allele Count** |
| --- | --- | --- | --- | --- | --- | --- | --- | --- |
|  |  |  | **RK** | **MK** | **EK** | **AK** |  |  |
| NT5en.1 | - | - | Het | Het | Hom | Het | rs1609407 | T=0.237908 (62972/264690, TOPMED) |
|  | - | - | Het | Het | Hom | Het | rs6947507 | T=0.266920 (70651/264690, TOPMED) |
| NT5en.2 |  |  | **No changes detected** | | | | | |
| NT5p.1 |  |  | **No changes in the distal promoter detected** | | | | | |
|  | - | - | Het | Het | Hom | Het | rs6976843 | T=0.301194 (79723/264690, TOPMED) |
|  | - | - | Het | Het | Hom | Het | rs10262141 | G=0.314345 (83204/264690, TOPMED) |
| NT5p.2.1 |  |  | **No changes in the proximal promoter detected** | | | | | |
|  | - | - | Het | Het | Hom | Het | rs13228827 | G=0.273671 (72438/264690, TOPMED) |
|  | - | - | Het | Het | Hom | Het | rs13228639 | C=0.314356 (83207/264690, TOPMED) |
| NT5utr5.1 |  |  | **No changes in exon 1 detected** | | | | | |
|  | - | - | Het | Het | Hom | Het | rs10230500 | T=0.273501 (72393/264690, TOPMED) |
|  | NM_001166118.3:c.-142+246C>T | - | Het | Het | Hom | Het | rs6948212 | G=0.273516 (72397/264690, TOPMED) |
| NT5utr5.2 |  |  | **No changes in exon 2 detected** | | | | | |
|  | NM_001166118.3:c. -141-37_-141-35del |  | Het | Het | Het | Het | rs201758497 | delA=0.00263 (44/16758, 8.3KJPN)  delA=0.0030 (15/5008, 1000G) |
| NT5utr5.R |  |  | **No changes detected** | | | | | |
| **GTTdel.w** | **NM_001166118.3: c.444_446del** | **NP_001361265.1: p.Phe149del** | **Het** | **Het** | **-** | **-** | **rs1227859962** | **delACA=0.000014 (2/140214, GnomAD)**  **delACA=0.00007 (1/15150, ALFA)**  **delACA=0.0002 (1/4480, Estonian)** |
| NT5utr.3 | **NC_000007.14:g.33014505_33014506insAAGA** NM_001166118.3: c.*224_*225= | - | Het | Het | Hom | Het | rs199721569 | -=0.274820 (72742/264690, TOPMED) |
|  | **NC_000007.14:g.33014500A>G** NM_001166118.3: c.*230= | - | Het | Het | Hom | Het | rs12536321 | A=0.273546 (72405/264690, TOPMED) |

**Table S1.15** Polymorphisms/mutations present in both brothers located in genes encoding known 5'-nucleotidases involved in pyrimidine and purine metabolism identified using the WES analysis of studied family members (het – heterozygotic; hom – homozygotic).

| **Chr** | **Position** | **Gene Symbol** | **Transcript Variant** | **Protein Variant** | **Genotype** | | | | **dbSNP ID** | **Frequency** |
| --- | --- | --- | --- | --- | --- | --- | --- | --- | --- | --- |
|  |  |  |  |  | **RK** | **MK** | **EK** | **AK** |  |  |
| 3 | 52558904 | *NT5DC2* | c.1348-7T>A; c.1237-7T>A |  | Het | Hom | Hom | Het | 34005367 | T=0.42667 (102123/239348, GnomAD)  T=0.37552 (47153/125568, TOPMED)  T=0.41624 (49170/118128, ExAC) |
| 3 | 52561779 | *NT5DC2* | c.825-45G>A; c.936-45G>A |  | Het | Hom | Hom | Het | 11711421 | T=0.44225 (108242/244754, GnomAD)  T=0.42022 (52766/125568, TOPMED)  T=0.43424 (51953/119640, ExAC) |
| 6 | 86180926 | *NT5E* | c.563-29A>G |  | Hom | Hom | Hom | Hom | 10944129 | A=0.46354 (89748/193614, GnomAD)  G=0.46576 (58485/125568, TOPMED)  A=0.3735 (22859/61206, ExAC) |
| 6 | 86199329 | *NT5E* | c.1210+12G>C |  | Hom | Hom | Hom | Hom | 9450284 | G=0.39274 (95080/242096, GnomAD)  G=0.46249 (58074/125568, TOPMED)  G=0.4797 (14807/30866, GnomAD) |
| 7 | 33057160 | *NT5C3A* | c.480_482delGTT; c.444_446delGTT; c.597_599delGTT | p.F149del; p.F200del; p.F161del | Het | Het |  |  | 1227859962 | delACA=0.0000 (1/30980, GnomAD)  delACA=0.000 (1/4480, Estonian) |
| 7 | 33059407 | *NT5C3A* | c.339-71T>C; c.303-71T>C; c.456-71T>C |  | Het | Het | Hom | Het | 2392209 | A=0.23370 (29345/125568, TOPMED)  A=0.2228 (6889/30924, GnomAD)  A=0.231 (1157/5008, 1000G) |
| 10 | 104851285 | *NT5C2* | c.1211+36C>T |  | Het | Het | Het | Hom | 10883830 | A=0.28515 (69553/243920, GnomAD)  A=0.30969 (38887/125568, TOPMED)  A=0.28852 (34897/120950, ExAC) |
| 10 | 104851396 | *NT5C2* | c.1160-24C>A |  | Het | Het | Het | Hom | 11191553 | T=0.28540 (70001/245270, GnomAD)  T=0.30970 (38889/125568, TOPMED)  T=0.28840 (34977/121278, ExAC) |
| 10 | 104855656 | *NT5C2* | c.813+40A>G |  | Het | Het | Het | Hom | 1926030 | C=0.42294 (103173/243944, GnomAD)  C=0.41544 (52166/125568, TOPMED)  C=0.42481 (51541/121328, ExAC) |
| 10 | 104855670 | *NT5C2* | c.813+26C>T |  | Het | Het | Het | Hom | 1926029 | A=0.28598 (69994/244752, GnomAD)  A=0.31023 (38955/125568, TOPMED)  A=0.28868 (35035/121362, ExAC) |
| 17 | 39987130 | *NT5C3B* | c.327A>G | p.A109A | Hom | Hom | Hom | Hom | 4796712 | T=0.08241 (19943/241998, GnomAD)  T=0.10108 (12693/125568, TOPMED)  T=0.1069 (3308/30946, GnomAD) |
| 17 | 39988752 | *NT5C3B* | c.229-23T>C |  | Het | Het | Het |  | 191474860 | G=0.00649 (1596/245992, GnomAD)  G=0.00358 (449/125568, TOPMED)  G=0.00670 (812/121110, ExAC) |
| 17 | 39991790 | *NT5C3B* | c.112-266G>A |  | Hom | Hom | Hom | Hom | 1319763 | C=0.22585 (28359/125568, TOPMED)  C=0.2544 (7850/30862, GnomAD)  C=0.216 (1082/5008, 1000G) |
| 17 | 39991867 | *NT5C3B* | c.111+243_111+244insGATA |  | Hom | Hom | Hom | Hom | 3039772 | -=0.1968 (2131/10830, ExAC)  -=0.216 (1082/5008, 1000G)  -=0.269 (1203/4480, Estonian) |
| 17 | 73126747 | *NT5C* | c.337-15delA; c.452-10delA |  | Hom | Hom | Hom | Hom | 10715821 | T=0.01980 (4874/246182, GnomAD)  T=0.08298 (10420/125568, TOPMED)  T=0.02470 (2998/121380, ExAC) |
| 17 | 73127217 | *NT5C* | c.276-17C>G |  | Het | Hom | Het | Hom | 3736075 | C=0.32102 (78114/243332, GnomAD)  C=0.29015 (36434/125568, TOPMED)  C=0.31679 (36583/115482, ExAC |

**Table S1.16** List of genes encoding known 5'-nucleotidases involved in pyrimidine and purine metabolism and their biological function.

| **Gene** | **Protein name / Subunit structure / Catalytic activity** | **Cofactor** |
| --- | --- | --- |
| ***NT5C3A*** | **Cytosolic 5'-nucleotidase 3A**  Monomer. Nucleotidase shows specific activity towards cytidine monophosphate (CMP) and 7-methylguanosine monophosphate (m_7_GMP). CMP seems to be the preferred substrate. [1–3] | Mg^2+^ |
| ***NT5C3B*** | **7-methylguanosine phosphate-specific 5'-nucleotidase**  Monomer. Specifically hydrolyzes 7-methylguanosine monophosphate (m_7_GMP) to 7-methylguanosine and inorganic phosphate. The specific activity for m_7_GMP may protect cells against undesired salvage of m_7_GMP and its incorporation into nucleic acids. Also has a weak activity for CMP. UMP and purine nucleotides are poor substrates. [2,4] | Mg^2+^ |
| ***NT5C2*** | **Cytosolic purine 5'-nucleotidase**  Homotetramer. May have a critical role in the maintenance of a constant composition of intracellular purine/pyrimidine nucleotides in cooperation with other nucleotidases. Preferentially hydrolyzes inosine 5'-monophosphate (IMP) and other purine nucleotides. [3] | Mg^2+^ |
| ***NT5C*** | **5'(3')-deoxyribonucleotidase, cytosolic type**  Homodimer. Dephosphorylates the 5' and 2'(3')-phosphates of deoxyribonucleotides, with a preference for dUMP and dTMP, intermediate activity towards dGMP, and low activity towards dCMP and dAMP. [5] | Mg^2+^ |
| ***NT5C1A*** | **Cytosolic 5'-nucleotidase 1A**  Dephosphorylates the 5' and 2'(3')-phosphates of deoxyribonucleotides and has a broad substrate specificity. Helps to regulate adenosine levels in the heart during ischemia and hypoxia. [6] | Mg^2+^ |
| ***NT5C1B*** | **Cytosolic 5'-nucleotidase 1B**  Dephosphorylates the 5' and 2'(3')-phosphates of deoxyribonucleotides. Helps to regulate adenosine levels (By similarity). | Mg^2+^ |
| ***NT5E*** | **5'-nucleotidase**  Homodimer. Hydrolyzes extracellular nucleotides into membrane permeable nucleosides. Exhibits AMP-, NAD-, and NMN-nucleosidase activities. [7,8] | Zn^2+^ |
| ***NT5M*** | **5'(3')-deoxyribonucleotidase, mitochondrial**  Homodimer. Dephosphorylates specifically the 5' and 2'(3')-phosphates of uracil and thymine deoxyribonucleotides, and so protect mitochondrial DNA replication from excess dTTP. Has only marginal activity towards dIMP and dGMP. [9,10] | Mg^2+^ |

1. Amici, A.; Ciccioli, K.; Naponelli, V.; Raffaelli, N.; Magni, G. Evidence for essential catalytic determinants for human erythrocyte pyrimidine 5′-nucleotidase. *Cellular and Molecular Life Sciences* **2005**, *62*, 1613–1620, doi:10.1007/s00018-005-5135-y.

2. Monecke, T.; Buschmann, J.; Neumann, P.; Wahle, E.; Ficner, R. Crystal Structures of the Novel Cytosolic 5′-Nucleotidase IIIB Explain Its Preference for m7GMP. *PLoS ONE* **2014**, *9*, e90915, doi:10.1371/journal.pone.0090915.

3. Walldén, K.; Stenmark, P.; Nyman, T.; Flodin, S.; Gräslund, S.; Loppnau, P.; Bianchi, V.; Nordlund, P. Crystal structure of human cytosolic 5′-nucleotidase II: Insights into allosteric regulation and substrate recognition. *Journal of Biological Chemistry* **2007**, *282*, 17828–17836, doi:10.1074/jbc.M700917200.

4. Buschmann, J.; Moritz, B.; Jeske, M.; Lilie, H.; Schierhorn, A.; Wahle, E. Identification of drosophila and human 7-methyl GMP-specific nucleotidases. *Journal of Biological Chemistry* **2013**, *288*, 2441–2451, doi:10.1074/jbc.M112.426700.

5. Walldén, K.; Rinaldo-Matthis, A.; Ruzzenente, B.; Rampazzo, C.; Bianchi, V.; Nordlund, P. Crystal Structures of Human and Murine Deoxyribonucleotidases: Insights into Recognition of Substrates and Nucleotide Analogues †. *Biochemistry* **2007**, *46*, 13809–13818, doi:10.1021/bi7014794.

6. Hunsucker, S.A.; Spychala, J.; Mitchell, B.S. Human Cytosolic 5′-Nucleotidase I. *Journal of Biological Chemistry* **2001**, *276*, 10498–10504, doi:10.1074/jbc.M011218200.

7. Garavaglia, S.; Bruzzone, S.; Cassani, C.; Canella, L.; Allegrone, G.; Sturla, L.; Mannino, E.; Millo, E.; De Flora, A.; Rizzi, M. The high-resolution crystal structure of periplasmic Haemophilus influenzae NAD nucleotidase reveals a novel enzymatic function of human CD73 related to NAD metabolism. *Biochemical Journal* **2012**, *441*, 131–141, doi:10.1042/BJ20111263.

8. Knapp, K.; Zebisch, M.; Pippel, J.; El-Tayeb, A.; Müller, C.E.; Sträter, N. Crystal Structure of the Human Ecto-5′-Nucleotidase (CD73): Insights into the Regulation of Purinergic Signaling. *Structure* **2012**, *20*, 2161–2173, doi:10.1016/j.str.2012.10.001.

9. Rampazzo, C.; Gallinaro, L.; Milanesi, E.; Frigimelica, E.; Reichard, P.; Bianchi, V. A deoxyribonucleotidase in mitochondria: involvement in regulation of dNTP pools and possible link to genetic disease. *Proceedings of the National Academy of Sciences of the United States of America* **2000**, *97*, 8239–44.

10. Rinaldo-Matthis, A.; Rampazzo, C.; Reichard, P.; Bianchi, V.; Nordlund, P. Crystal structure of a human mitochondrial deoxyribonucleotidase. *Nature Structural Biology* **2002**, doi:10.1038/nsb846.
