# Supplementary material for: A rare mutation (p.F149del) of the NT5C3A gene is associated with pyrimidine 5′-nucleotidase deficiency
Source: Cell Mol Biol Lett. 2022 Nov 24;27:104. doi: 10.1186/s11658-022-00405-w (PMC9700897; doi:10.1186/s11658-022-00405-w)
Supplement: Supplementary file 4 — Additional file 4: Supplementary raw WB data. [file 11658_2022_405_MOESM4_ESM.pdf]

Supplementary raw WB data

CMBL_Boguslawskaetal_NT5C3A_figure 3 D – uncropedWB

Replicate 1

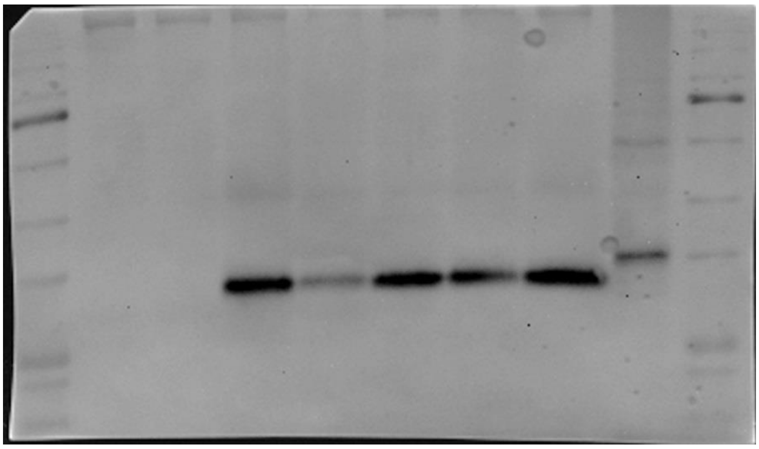

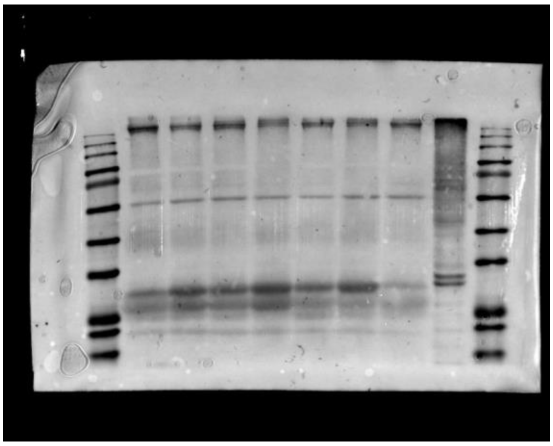

15.03.2018
12% gel, 1mm, 25 mA, 2h
I°Ab mouse anti-NT5C3 sc-390782 (SCBT, 1:1000), 60sec

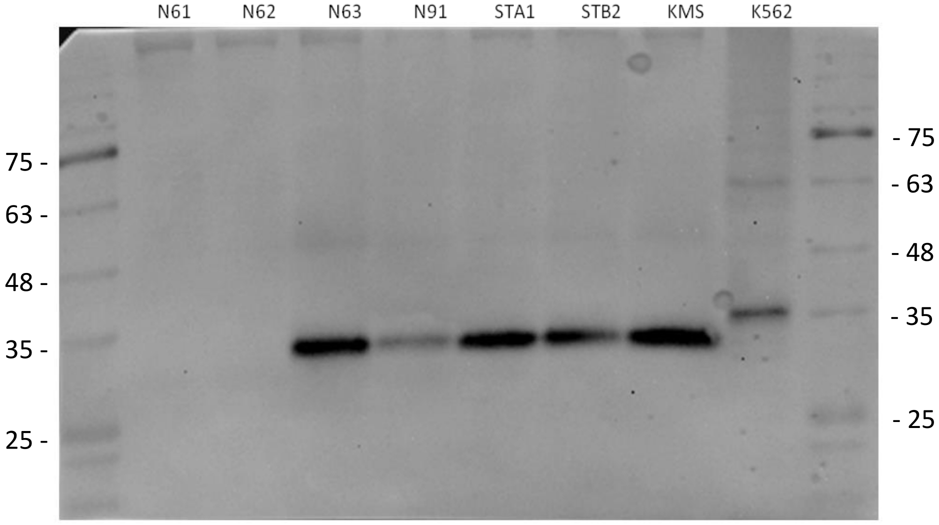

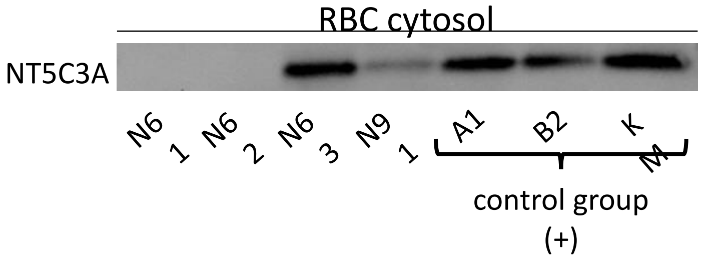

Replicate 2

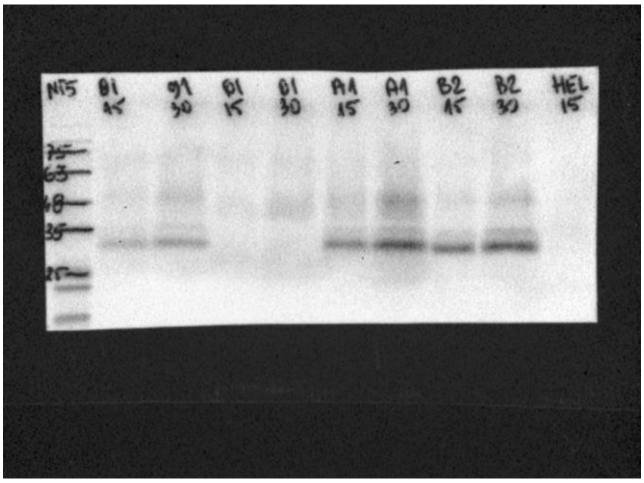

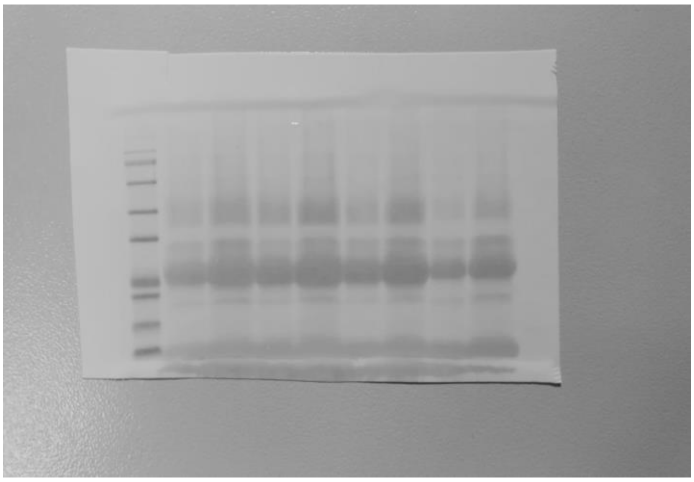

NT5C3A **Total protein**

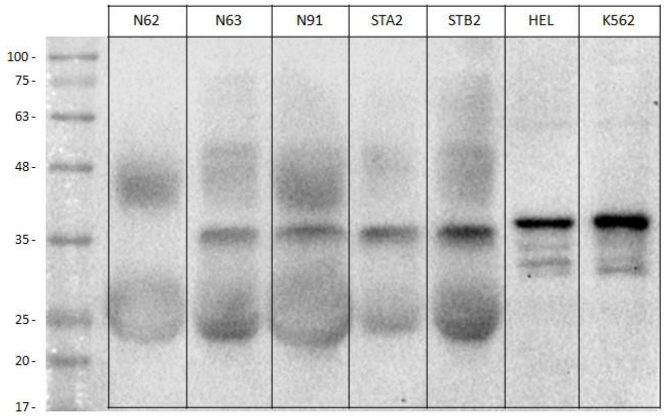

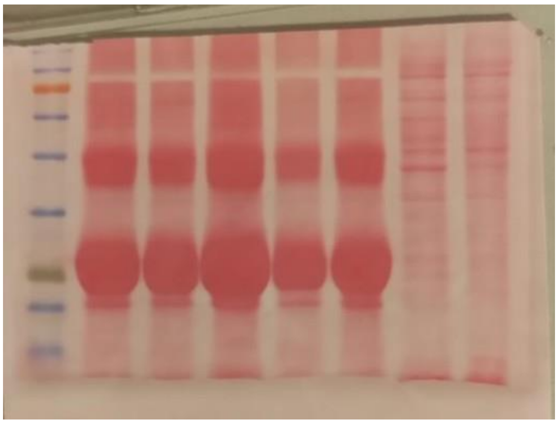

NT5C3A **Total protein**
